# Supplementary material for: Conversion from coniferous to broadleaved trees can make European forests more climate-effective
Source: Nat Commun. 2025 Oct 29;16:9536. doi: 10.1038/s41467-025-64580-y (PMC12572288; doi:10.1038/s41467-025-64580-y)
Supplement: Supplementary file 1 — Supplementary Information [file 41467_2025_64580_MOESM1_ESM.pdf]

Supplementary Information - Conversion from coniferous to broadleaved trees can make European forests more climate-effective

Yao et al.

Contents

List of Figures **3**

1 Supplementary Figures referred to in the manuscript **9**

2 Supplementary Tables referred to in the manuscript **23**

3 Supplementary Figures of other temperatures **29**

4 Supplementary Notes **41**

4.1 Supplementary Note 1: Evaluation of COSMO – CLM^2 performance 41

4.2 Supplementary Note 2: Results from additional sensitivity tests 45

Supplementary References **47**

List of Figures

S1 Multi-year (2025-2059) summer (June, July, and August) mean up-welling shortwave radiation ($SW_{up,jjaM}$: **a**), down-welling shortwave radiation ($SW_{down,jjaM}$: **e**), and net shortwave radiation ($SW_{net,jjaM}$: **i**), under the present-day forest scenario (Ctl). Changes in these radiation fluxes under the conversion from coniferous to broadleaf forests scenario (Brd-Ctl: **b,f,j**), under the deforestation scenario (Def-Ctl: **c,g,k**), and the difference between the two scenarios (Brd-Def: **d,h,l**). . . . 9

S2 Multi-year (2025-2059) summer (June, July, and August) mean latent heat flux from the land to the atmosphere ($LE_{up,jjaM}$: **a**), down-welling longwave radiation ($LW_{down,jjaM}$: **e**), sensible heat flux from the land to the atmosphere ($H_{up,jjaM}$: **i**), the ground flux (from the land surface to the ground, $G_{down,jjaM}$: **m**), and up-welling longwave radiation ($LW_{up,jjaM}$: **q**), under the present-day forest scenario (Ctl). Changes in these energy fluxes under the conversion from coniferous to broadleaf forests scenario (Brd-Ctl: **b,f,j,n,r**), by the deforestation scenario (Def-Ctl: **c,g,k,o,s**), and the difference between the two scenarios (Brd-Def: **d,h,l,p,t**). 10

S3 Multi-year (2025-2059) summer (June, July, and August) mean daily mean land surface temperature ($T_{sfc,jjaM}$: **a**), 2-meter air temperature ($T_{air,jjaM}$: **e**), and the temperature at the lowest atmospheric level ($T_{atm,jjaM}$: **i**), under the present-day forest scenario (Ctl). Changes in these temperatures under the conversion from coniferous to broadleaf forests scenario (Brd-Ctl: **b,f,j**), under the deforestation scenario (Def-Ctl: **c,g,k**), and the difference between the two scenarios (Brd-Def: **d,h,l**). 11

S4 Multi-year (2025-2059) summer (June, July, and August) mean daily minimum land surface temperature ($T_{sfc,jjaN}$: **a**), 2-meter air temperature ($T_{air,jjaN}$: **e**), and the temperature at the lowest atmospheric level ($T_{atm,jjaN}$: **i**), under the present-day forest scenario (Ctl). Changes in these temperatures under the conversion from coniferous to broadleaf forests scenario (Brd-Ctl: **b,f,j**), under the deforestation scenario (Def-Ctl: **c,g,k**), and the difference between the two scenarios (Brd-Def: **d,h,l**). 12

S5 Changes in multi-year mean (2025-2059) monthly mean daily mean temperature (T_{monM}) induced by the conversion from coniferous to broadleaf forests scenario (Brd-Ctl) (**left column**) and the deforestation scenario (Def-Ctl) (**right column**). The values shown are the regionally averaged temperature change (minus the outputs from the Ctl simulation) over five regions: Alpine (**a-b**), Northern (**c-d**), Atlantic (**e-f**), Continental (**g-h**), and Southern (**i-j**). 13

S6 Changes in multi-year mean (2025-2059) monthly mean daily minimum temperature (T_{monN}) induced by the conversion from coniferous to broadleaf forests scenario (Brd-Ctl) (**left column**) and the deforestation scenario (Def-Ctl) (**right column**). The values shown are the regionally averaged temperature change (minus the outputs from the Ctl simulation) over five regions: Alpine (**a-b**), Northern (**c-d**), Atlantic (**e-f**), Continental (**g-h**), and Southern (**i-j**). 14

S7 Changes in multi-year mean (2025-2059) monthly mean daily mean up-welling longwave radiation (LW_{up}), sensible heat flux from the land to the atmosphere (H_{up}), net shortwave radiation (SW_{net}), down-welling longwave radiation (LW_{down}), ground energy flux from the land surface to the ground (G_{down}), and latent heat flux from the land to the atmosphere (LE_{up}), induced by the conversion from coniferous to broadleaf forests scenario (Brd) (**left column**) and the deforestation scenario (Def) (**right column**). The values shown are the regionally averaged temperature change (minus the outputs from the Ctl simulation) over five regions: Alpine (**a-b**), Northern (**c-d**), Atlantic (**e-f**), Continental (**g-h**), and Southern (**i-j**). 15

S8 Changes in multi-year (2025-2059, compared to the experiment Ctl) summer (June, July, and August) mean daily mean land surface temperature ($T_{sfc,jjaM}$: **a-d**), 2-meter air temperature ($T_{air,jjaM}$: **e-h**), and the temperature at the lowest atmospheric level ($T_{atm,jjaM}$: **i-l**) under the forestation scenario (Aff-Ctl: **a,e,i**), under the combining forestation and conversion from coniferous to broadleaf forests scenario (AfB-Ctl: **b,f,j**), the difference between the two scenarios (AfB-Aff: **c,g,k**), and the difference between AfB and the deforestation scenario (AfB-Def: **d,h,l**). 16

S9 Changes in multi-year (2025-2059, compared to the experiment Ctl) summer (June, July, and August) mean daily minimum land surface temperature ($T_{sfc,jjaN}$: **a-d**), 2-meter air temperature ($T_{air,jjaN}$: **e-h**), and the temperature at the lowest atmospheric level ($T_{atm,jjaN}$: **i-l**) under the forestation scenario (Aff-Ctl: **a,e,i**), under the combining forestation and conversion from coniferous to broadleaf forests scenario (AfB-Ctl: **b,f,j**), the difference between the two scenarios (AfB-Aff: **c,g,k**), and the difference between AfB and the deforestation scenario (AfB-Def: **d,h,l**). 17

S10 Changes in multi-year (2025-2059, compared to the experiment Ctl) summer (June, July, and August) mean up-welling shortwave radiation ($SW_{up,jjaM}$: **a-d**), down-welling shortwave radiation ($SW_{down,jjaM}$: **e-h**), and net shortwave radiation ($SW_{net,jjaM}$: **i-l**) under the forestation scenario (Aff-Ctl: **a,e,i**), under the combining forestation and conversion from coniferous to broadleaf forests scenario (AfB-Ctl: **b,f,j**), the difference between the two scenarios (AfB-Aff: **c,g,k**), and the difference between AfB and the deforestation scenario (AfB-Def: **d,h,l**). 18

S11 Changes in multi-year (2025-2059, compared to the experiment Ctl) summer (June, July, and August) mean latent heat flux from the land to the atmosphere ($LE_{up,jjaM}$: **a-d**), down-welling longwave radiation ($LW_{down,jjaM}$: **e-h**), sensible heat flux from the land to the atmosphere ($H_{up,jjaM}$: **i-l**), the ground flux (from the land surface to the ground, $G_{down,jjaM}$: **m-p**), and up-welling longwave radiation ($LW_{up,jjaM}$: **q-t**). Changes in these fluxes under the forestation scenario (Aff-Ctl: **a,e,i,m,q**), by the combining scenario of forestation and the conversion from coniferous to broadleaf forests (AfB-Ctl: **b,f,j,n,r**), the difference between the two scenarios (AfB-Aff: **c,g,k,o,s**), and the difference between AfB and the deforestation scenario (AfB-Def: **d,h,l,p,t**). 19
S12 Changes in multi-year mean (2025-2059) monthly mean daily mean temperature (T_{monM}) induced by the forestation scenario (Aff) (**left column**) and the combining scenario of forestation and the conversion from coniferous to broadleaf forests (AfB) (**right column**). The values shown are the regionally averaged temperature change (minus the outputs from the Ctl simulation) over five regions: Alpine (**a-b**), Northern (**c-d**), Atlantic (**e-f**), Continental (**g-h**), and Southern (**i-j**). . . 20
S13 Changes in multi-year mean (2025-2059) monthly mean daily minimum temperature (T_{monN}) induced by the forestation scenario (Aff) (**left column**) and the combining scenario of forestation and the conversion from coniferous to broadleaf forests (AfB) (**right column**). The values shown are the regionally averaged temperature change (minus the outputs from the Ctl simulation) over five regions: Alpine (**a-b**), Northern (**c-d**), Atlantic (**e-f**), Continental (**g-h**), and Southern (**i-j**). 21
S14 Changes in multi-year mean (2025-2059) monthly mean daily mean up-welling longwave radiation (LW_{up}), sensible heat flux from the land to the atmosphere (H_{up}), net shortwave radiation (SW_{net}), down-welling longwave radiation (LW_{down}), ground energy flux from the land surface to the ground (G_{down}), and latent heat flux from the land to the atmosphere (LE_{up}), induced by the forestation scenario (Aff) (**left column**) and the combining scenario of forestation and the conversion from coniferous to broadleaf forests (AfB) (**right column**). The values shown are the regionally averaged temperature change (minus the outputs from the Ctl simulation) over five regions: Alpine (**a-b**), Northern (**c-d**), Atlantic (**e-f**), Continental (**g-h**), and Southern (**i-j**). 22
S15 Multi-year (2025-2059) annual mean daily mean land surface temperature ($T_{sfc,yearM}$: **a**), 2-meter air temperature ($T_{air,yearM}$: **e**), and the temperature at the lowest atmospheric level ($T_{atm,yearM}$: **i**), under the present-day forest scenario (Ctl). Changes in these temperatures under the conversion from coniferous to broadleaf forests scenario (Brd-Ctl: **b,f,j**), by the deforestation scenario (Def-Ctl: **c,g,k**), and the difference between the two scenarios (Brd-Def: **d,h,l**). 29

S16 Multi-year (2025-2059) annual mean daily maximum land surface temperature ($T_{sfc,yearX}$: **a**), 2-meter air temperature ($T_{air,yearX}$: **e**), and the temperature at the lowest atmospheric level ($T_{atm,yearX}$: **i**), under the present-day forest scenario (Ctl). Changes in these temperatures under the conversion from coniferous to broadleaf forests scenario (Brd-Ctl: **b,f,j**), by the deforestation scenario (Def-Ctl: **c,g,k**), and the difference between the two scenarios (Brd-Def: **d,h,l**). . . . 30

S17 Multi-year (2025-2059) annual mean daily maximum land surface temperature ($T_{sfc,yearN}$: **a**), 2-meter air temperature ($T_{air,yearN}$: **e**), and the temperature at the lowest atmospheric level ($T_{atm,yearN}$: **i**), under the present-day forest scenario (Ctl). Changes in these temperatures under the conversion from coniferous to broadleaf forests scenario (Brd-Ctl: **b,f,j**), by the deforestation scenario (Def-Ctl: **c,g,k**), and the difference between the two scenarios (Brd-Def: **d,h,l**). . . . 31

S18 Multi-year (2025-2059) summer (March, April, and May) mean daily mean land surface temperature ($T_{sfc,mamM}$: **a**), 2-meter air temperature ($T_{air,mamM}$: **e**), and the temperature at the lowest atmospheric level ($T_{atm,mamM}$: **i**), under the present-day forest scenario (Ctl). Changes in these temperatures under the conversion from coniferous to broadleaf forests scenario (Brd-Ctl: **b,f,j**), by the deforestation scenario (Def-Ctl: **c,g,k**), and the difference between the two scenarios (Brd-Def: **d,h,l**). 32

S19 Multi-year (2025-2059) summer (March, April, and May) mean daily maximum land surface temperature ($T_{sfc,mamX}$: **a**), 2-meter air temperature ($T_{air,mamX}$: **e**), and the temperature at the lowest atmospheric level ($T_{atm,mamX}$: **i**), under the present-day forest scenario (Ctl). Changes in these temperatures under the conversion from coniferous to broadleaf forests scenario (Brd-Ctl: **b,f,j**), by the deforestation scenario (Def-Ctl: **c,g,k**), and the difference between the two scenarios (Brd-Def: **d,h,l**). 33

S20 Multi-year (2025-2059, in the experiment Ctl) summer (March, April, and May) mean daily maximum land surface temperature ($T_{sfc,mamN}$: **a**), 2-meter air temperature ($T_{air,mamN}$: **e**), and the temperature at the lowest atmospheric level ($T_{atm,mamN}$: **i**). Changes in these temperatures under the conversion from coniferous to broadleaf forests scenario (Brd-Ctl: **b,f,j**), by the deforestation scenario (Def-Ctl: **c,g,k**), and the difference between the two scenarios (Brd-Def: **d,h,l**). 34

S21 Changes in multi-year (2025-2059, compared to the experiment Ctl) annual mean daily mean land surface temperature ($T_{sfc,yearM}$: **a-d**), 2-meter air temperature ($T_{air,yearM}$: **e-h**), and the temperature at the lowest atmospheric level ($T_{atm,yearM}$: **i-l**) under the forestation scenario (Aff-Ctl: **a,e,i**), by the combining forestation and conversion from coniferous to broadleaf forests scenario (AfB-Ctl: **b,f,j**), the difference between the two scenarios (Brd-Def: **c,g,k**), and the difference between AfB and the deforestation scenario (AfB-Def: **d,h,l**). 35

S22 Changes in multi-year (2025-2059, compared to the experiment Ctl) annual mean daily maximum land surface temperature ($T_{sfc,yearX}$: **a-d**), 2-meter air temperature ($T_{air,yearX}$: **e-h**), and the temperature at the lowest atmospheric level ($T_{atm,yearX}$: **i-l**) under the forestation scenario (Aff-Ctl: **a,e,i**), by the combining forestation and conversion from coniferous to broadleaf forests scenario (AfB-Ctl: **b,f,j**), the difference between the two scenarios (Brd-Def: **c,g,k**), and the difference between AfB and the deforestation scenario (AfB-Def: **d,h,l**). 36
S23 Changes in multi-year (2025-2059, compared to the experiment Ctl) annual mean daily minimum land surface temperature ($T_{sfc,yearN}$: **a-d**), 2-meter air temperature ($T_{air,yearN}$: **e-h**), and the temperature at the lowest atmospheric level ($T_{atm,yearN}$: **i-l**) under the forestation scenario (Aff-Ctl: **a,e,i**), by the combining forestation and conversion from coniferous to broadleaf forests scenario (AfB-Ctl: **b,f,j**), the difference between the two scenarios (Brd-Def: **c,g,k**), and the difference between AfB and the deforestation scenario (AfB-Def: **d,h,l**). 37
S24 Changes in multi-year (2025-2059, compared to the experiment Ctl) spring (March, April, and May) mean daily mean land surface temperature ($T_{sfc,mamM}$: **a-d**), 2-meter air temperature ($T_{air,mamM}$: **e-h**), and the temperature at the lowest atmospheric level ($T_{atm,mamM}$: **i-l**) under the forestation scenario (Aff-Ctl: **a,e,i**), by the combining forestation and conversion from coniferous to broadleaf forests scenario (AfB-Ctl: **b,f,j**), the difference between the two scenarios (Brd-Def: **c,g,k**), and the difference between AfB and the deforestation scenario (AfB-Def: **d,h,l**). 38
S25 Changes in multi-year (2025-2059, compared to the experiment Ctl) spring (March, April, and May) mean daily maximum land surface temperature ($T_{sfc,mamX}$: **a-d**), 2-meter air temperature ($T_{air,mamX}$: **e-h**), and the temperature at the lowest atmospheric level ($T_{atm,mamX}$: **i-l**) under the forestation scenario (Aff-Ctl: **a,e,i**), by the combining forestation and conversion from coniferous to broadleaf forests scenario (AfB-Ctl: **b,f,j**), the difference between the two scenarios (Brd-Def: **c,g,k**), and the difference between AfB and the deforestation scenario (AfB-Def: **d,h,l**). 39
S26 Changes in multi-year (2025-2059, compared to the experiment Ctl) spring (March, April, and May) mean daily minimum land surface temperature ($T_{sfc,mamN}$: **a-d**), 2-meter air temperature ($T_{air,mamN}$: **e-h**), and the temperature at the lowest atmospheric level ($T_{atm,mamN}$: **i-l**) under the forestation scenario (Aff-Ctl: **a,e,i**), by the combining forestation and conversion from coniferous to broadleaf forests scenario (AfB-Ctl: **b,f,j**), the difference between the two scenarios (Brd-Def: **c,g,k**), and the difference between AfB and the deforestation scenario (AfB-Def: **d,h,l**). 40
S27 **a,c** Simulated difference in multi-year (2025-2059) mean daily maximum (T_{skin}^{max}) and daily minimum land surface temperature (T_{skin}^{min}) between the forest and grassland land-use tile in the control simulation. **b,d** Observation-based difference in multi-year (2002-2012) mean T_{skin}^{max} and T_{skin}^{min}[4]. 42

S28 **a-e, k-o** Simulated difference in multi-year (2025-2059) mean daily maximum (T_{skin}^{max}) and daily minimum land surface temperature (T_{skin}^{min}) between the forestation and deforestation scenarios (Def-Aff). **b-f,p-t** Observation-based potential of changes in multi-year (2008-2012) mean T_{skin}^{max} and T_{skin}^{min} because of deforestation[5]. 42

S29 **a-e, k-o** Simulated difference in multi-year (2025-2059) mean daily maximum (T_{skin}^{max}) and daily minimum land surface temperature (T_{skin}^{min}) between the combining scenario of forestation and transition from broadleaf to coniferous forests and the opposite transition (AfN-AfB). **b-f,p-t** Observation-based potential of changes in multi-year (2008-2012) mean T_{skin}^{max} and T_{skin}^{min} because of the transition from broadleaf to coniferous forests[5]. 43

S30 **a-e, k-o** Simulated difference in multi-year (2025-2059) mean daily maximum (T_{skin}^{max}) and daily minimum land surface temperature (T_{skin}^{min}) between the coniferous forest and grassland land-use tiles, and between the broadleaf forest and grassland land-use tiles. **b-f,p-t** Calculated radiative impacts of the land-use change from grassland to coniferous or broadleaf forest[6]. 44

S31 **a-e** Simulated difference in multi-year (2025-2059) mean daily maximum (T_{skin}^{max}) and daily minimum land surface temperature (T_{skin}^{min}) between the broadleaf and coniferous land-use tiles. **b-f** Calculated radiative impacts of the land-use change from broadleaf to coniferous forest[6]. 44

S32 Changes in annual (T_{annM}), summer (T_{sumM}), and spring (T_{sprM}) mean air temperature by changes in canopy height and leaf area index (HGT^+: Canopy heights multiplied by 1.5; HGT^-: Canopy heights divided by 1.5; LAI^+: LAI multiplied by 1.5; LAI^-: LAI divided by 1.5; H^+L^+: both canopy heights and LAI multiplied by 1.5; H^-L^-: both canopy heights and LAI divided by 1.5) scenarios. 46

1 Supplementary Figures referred to in the manuscript

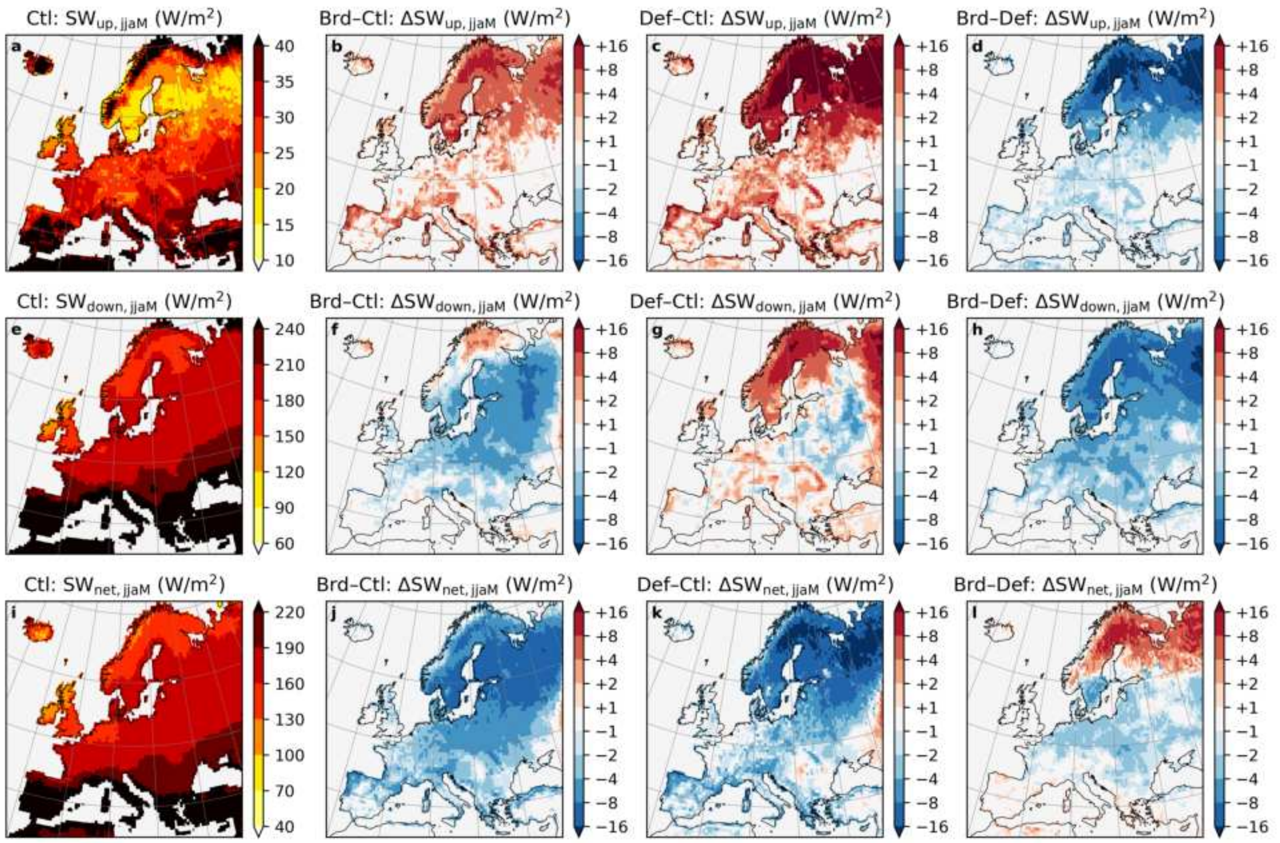

Supplementary Figure S1 | Multi-year (2025-2059) summer (June, July, and August) mean up-welling shortwave radiation ($SW_{up,jjaM}$: **a**), down-welling shortwave radiation ($SW_{down,jjaM}$: **e**), and net shortwave radiation ($SW_{net,jjaM}$: **i**), under the present-day forest scenario (Ctl). Changes in these radiation fluxes under the conversion from coniferous to broadleaf forests scenario (Brd-Ctl: **b,f,j**), under the deforestation scenario (Def-Ctl: **c,g,k**), and the difference between the two scenarios (Brd-Def: **d,h,l**).

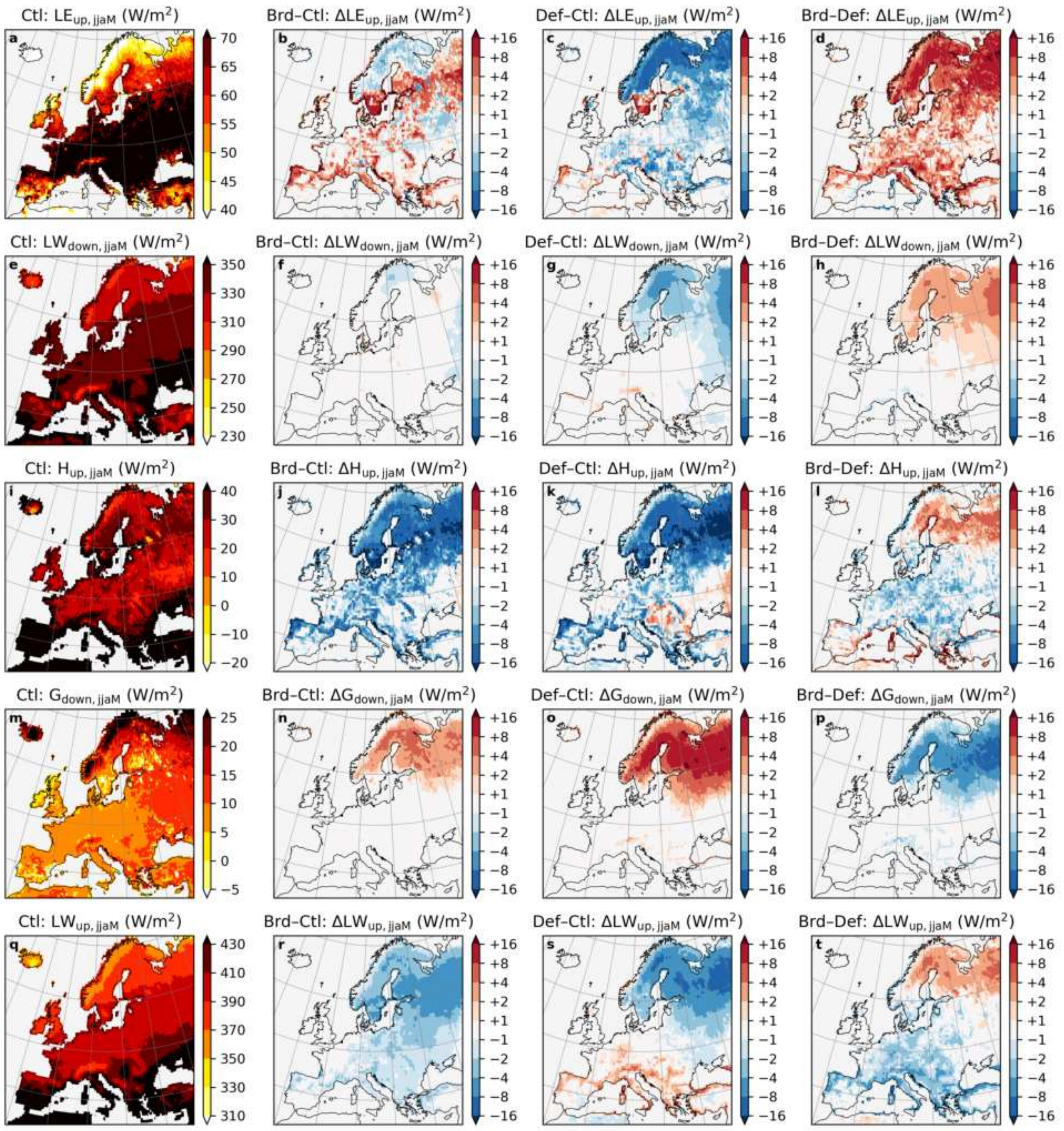

Supplementary Figure S2 | Multi-year (2025-2059) summer (June, July, and August) mean latent heat flux from the land to the atmosphere ($LE_{up,jjaM}$: **a**), down-welling longwave radiation ($LW_{down,jjaM}$: **e**), sensible heat flux from the land to the atmosphere ($H_{up,jjaM}$: **i**), the ground flux (from the land surface to the ground, $G_{down,jjaM}$: **m**), and up-welling longwave radiation ($LW_{up,jjaM}$: **q**), under the present-day forest scenario (Ctl). Changes in these energy fluxes under the conversion from coniferous to broadleaf forests scenario (Brd-Ctl: **b,f,j,n,r**), by the deforestation scenario (Def-Ctl: **c,g,k,o,s**), and the difference between the two scenarios (Brd-Def: **d,h,l,p,t**).

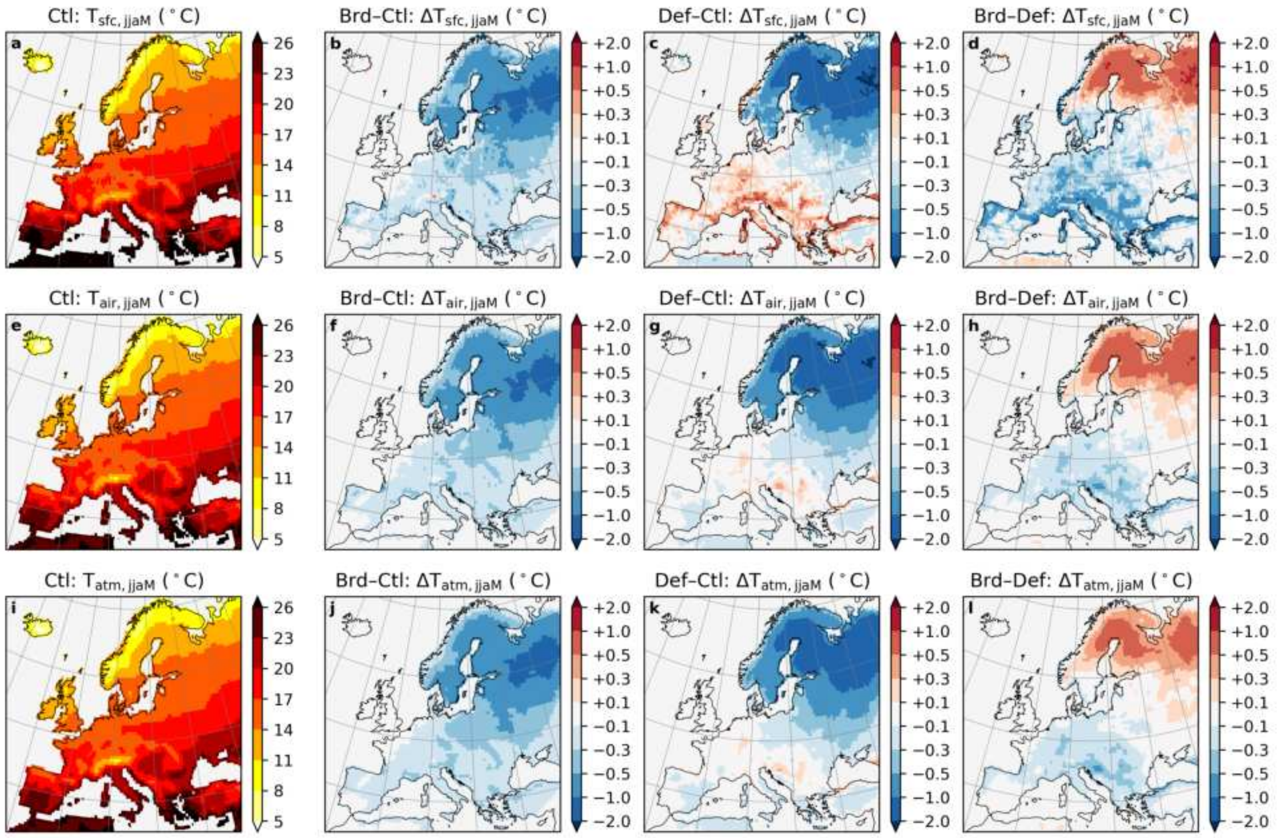

Supplementary Figure S3 | Multi-year (2025-2059) summer (June, July, and August) mean daily mean land surface temperature ($T_{sfc,jjaM}$: **a**), 2-meter air temperature ($T_{air,jjaM}$: **e**), and the temperature at the lowest atmospheric level ($T_{atm,jjaM}$: **i**), under the present-day forest scenario (Ctl). Changes in these temperatures under the conversion from coniferous to broadleaf forests scenario (Brd-Ctl: **b,f,j**), under the deforestation scenario (Def-Ctl: **c,g,k**), and the difference between the two scenarios (Brd-Def: **d,h,l**).

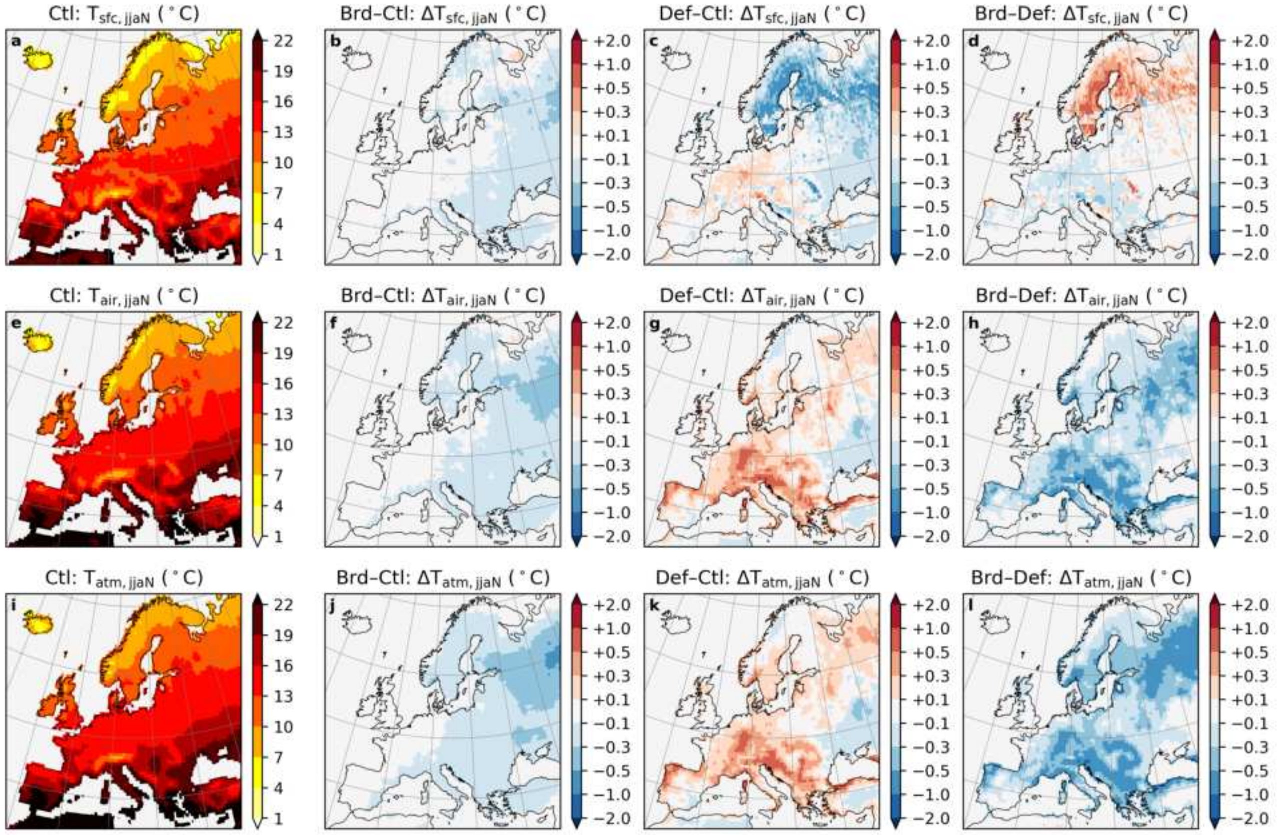

Supplementary Figure S4 | Multi-year (2025-2059) summer (June, July, and August) mean daily minimum land surface temperature ($T_{sfc,jjaN}$: **a**), 2-meter air temperature ($T_{air,jjaN}$: **e**), and the temperature at the lowest atmospheric level ($T_{atm,jjaN}$: **i**), under the present-day forest scenario (Ctl). Changes in these temperatures under the conversion from coniferous to broadleaf forests scenario (Brd-Ctl: **b,f,j**), under the deforestation scenario (Def-Ctl: **c,g,k**), and the difference between the two scenarios (Brd-Def: **d,h,l**).

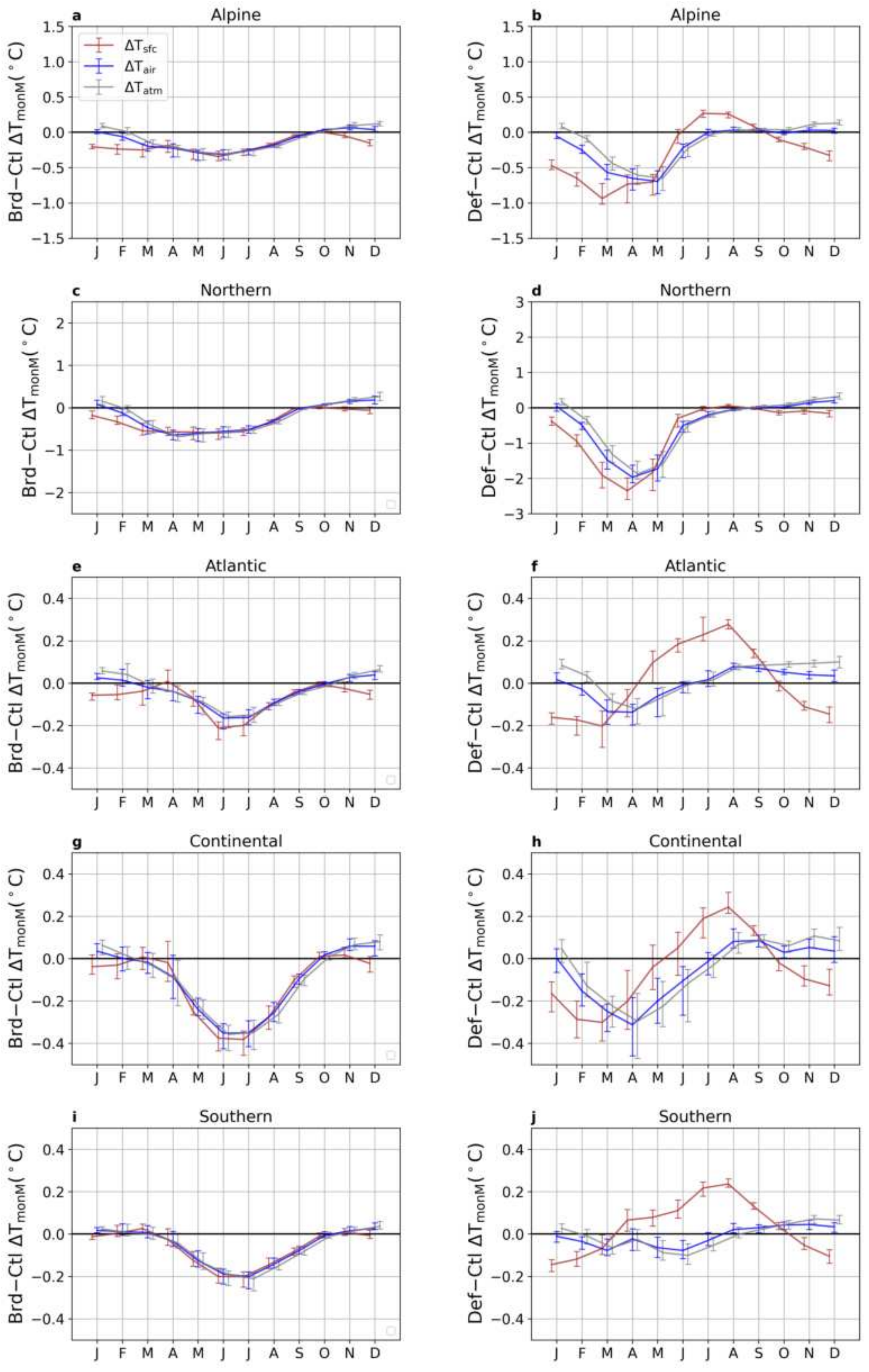

Supplementary Figure S5 | Changes in multi-year mean (2025-2059) monthly mean daily mean temperature (T_{monM}) induced by the conversion from coniferous to broadleaf forests scenario (Brd-Ctl) (**left column**) and the deforestation scenario (Def-Ctl) (**right column**). The values shown are the regionally averaged temperature change (minus the outputs from the Ctl simulation) over five regions: Alpine (**a-b**), Northern (**c-d**), Atlantic (**e-f**), Continental (**g-h**), and Southern (**i-j**).

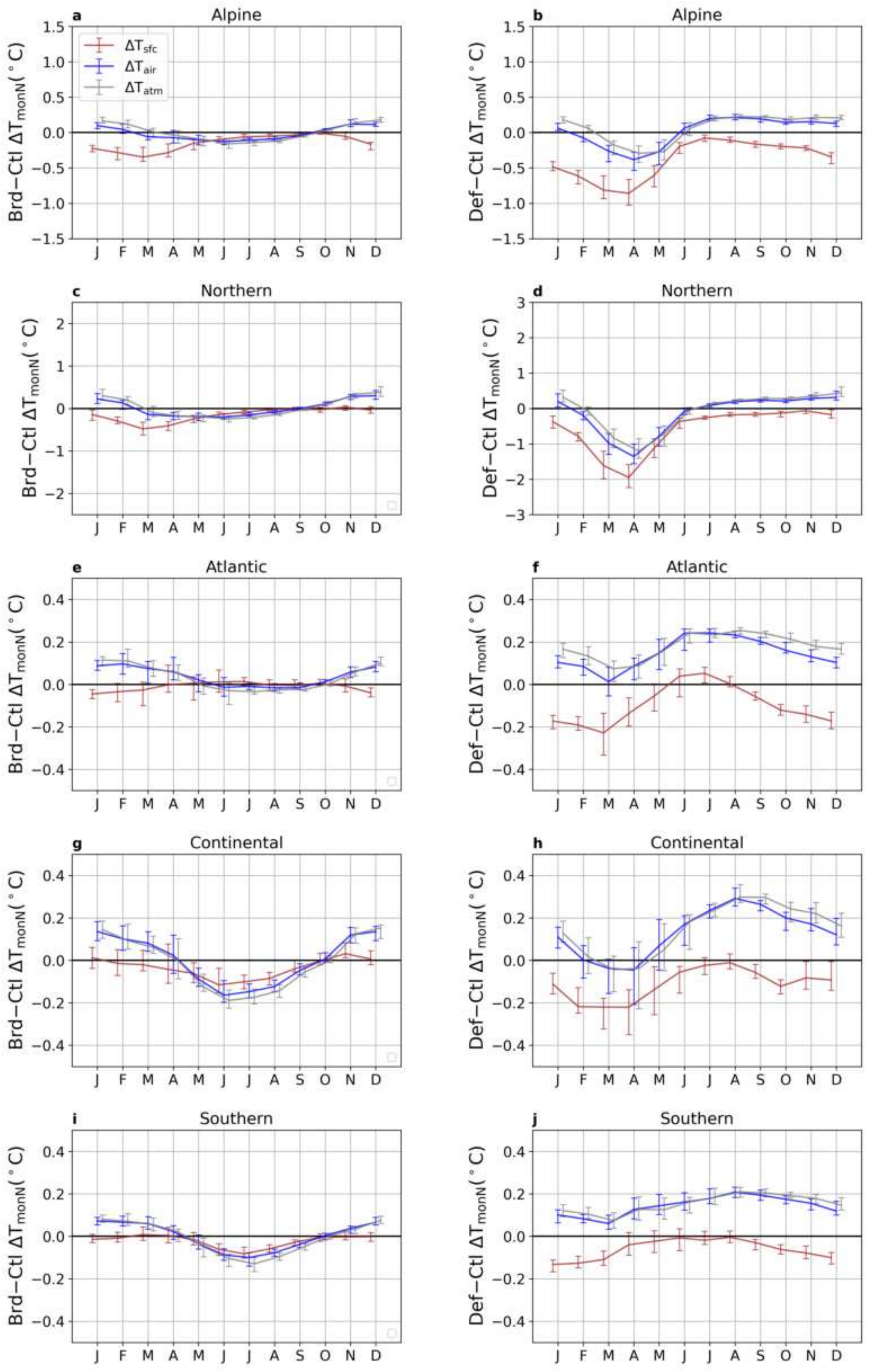

Supplementary Figure S6 | Changes in multi-year mean (2025-2059) monthly mean daily minimum temperature (T_{monN}) induced by the conversion from coniferous to broadleaf forests scenario (Brd-Ctl) (**left column**) and the deforestation scenario (Def-Ctl) (**right column**). The values shown are the regionally averaged temperature change (minus the outputs from the Ctl simulation) over five regions: Alpine (**a-b**), Northern (**c-d**), Atlantic (**e-f**), Continental (**g-h**), and Southern (**i-j**).

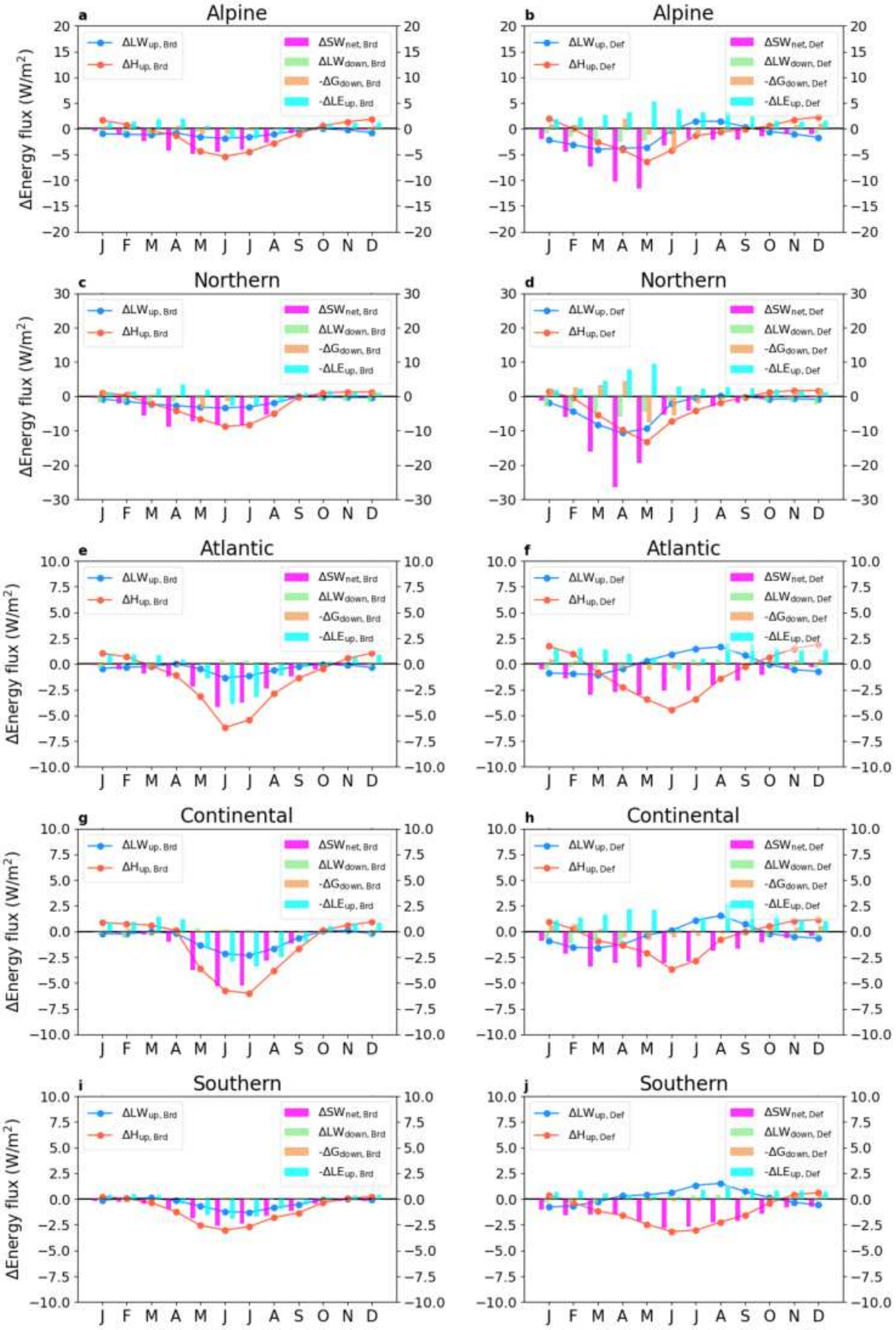

Supplementary Figure S7 | Changes in multi-year mean (2025-2059) monthly mean daily mean up-welling longwave radiation (LW_{up}), sensible heat flux from the land to the atmosphere (H_{up}), net shortwave radiation (SW_{net}), down-welling longwave radiation (LW_{down}), ground energy flux from the land surface to the ground (G_{down}), and latent heat flux from the land to the atmosphere (LE_{up}), induced by the conversion from coniferous to broadleaf forests scenario (Brd) (**left column**) and the deforestation scenario (Def) (**right column**). The values shown are the regionally averaged temperature change (minus the outputs from the Ctl simulation) over five regions: Alpine (**a-b**), Northern (**c-d**), Atlantic (**e-f**), Continental (**g-h**), and Southern (**i-j**).

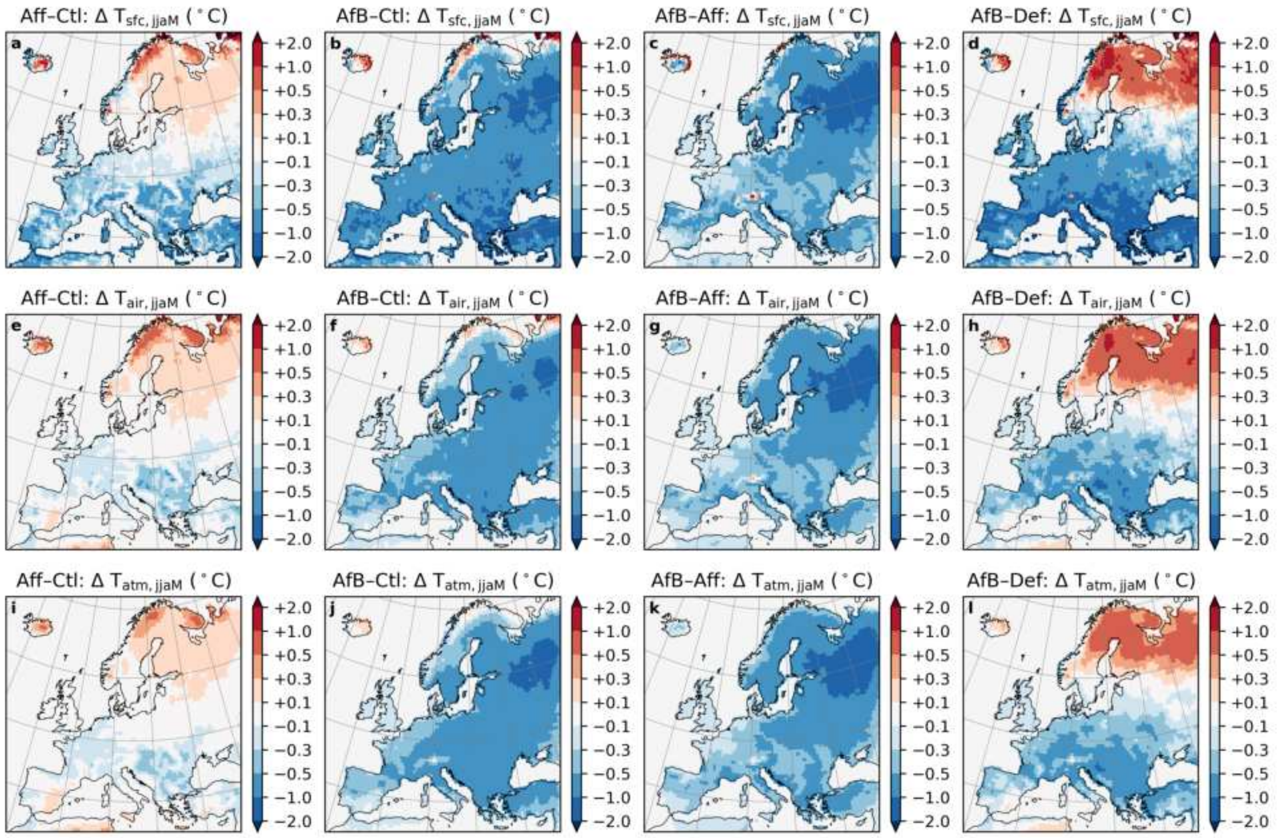

Supplementary Figure S8 | Changes in multi-year (2025-2059, compared to the experiment Ctl) summer (June, July, and August) mean daily mean land surface temperature ($T_{sfc,jjaM}$: **a-d**), 2-meter air temperature ($T_{air,jjaM}$: **e-h**), and the temperature at the lowest atmospheric level ($T_{atm,jjaM}$: **i-l**) under the forestation scenario (Aff-Ctl: **a,e,i**), under the combining forestation and conversion from coniferous to broadleaf forests scenario (AfB-Ctl: **b,f,j**), the difference between the two scenarios (AfB-Aff: **c,g,k**), and the difference between AfB and the deforestation scenario (AfB-Def: **d,h,l**).

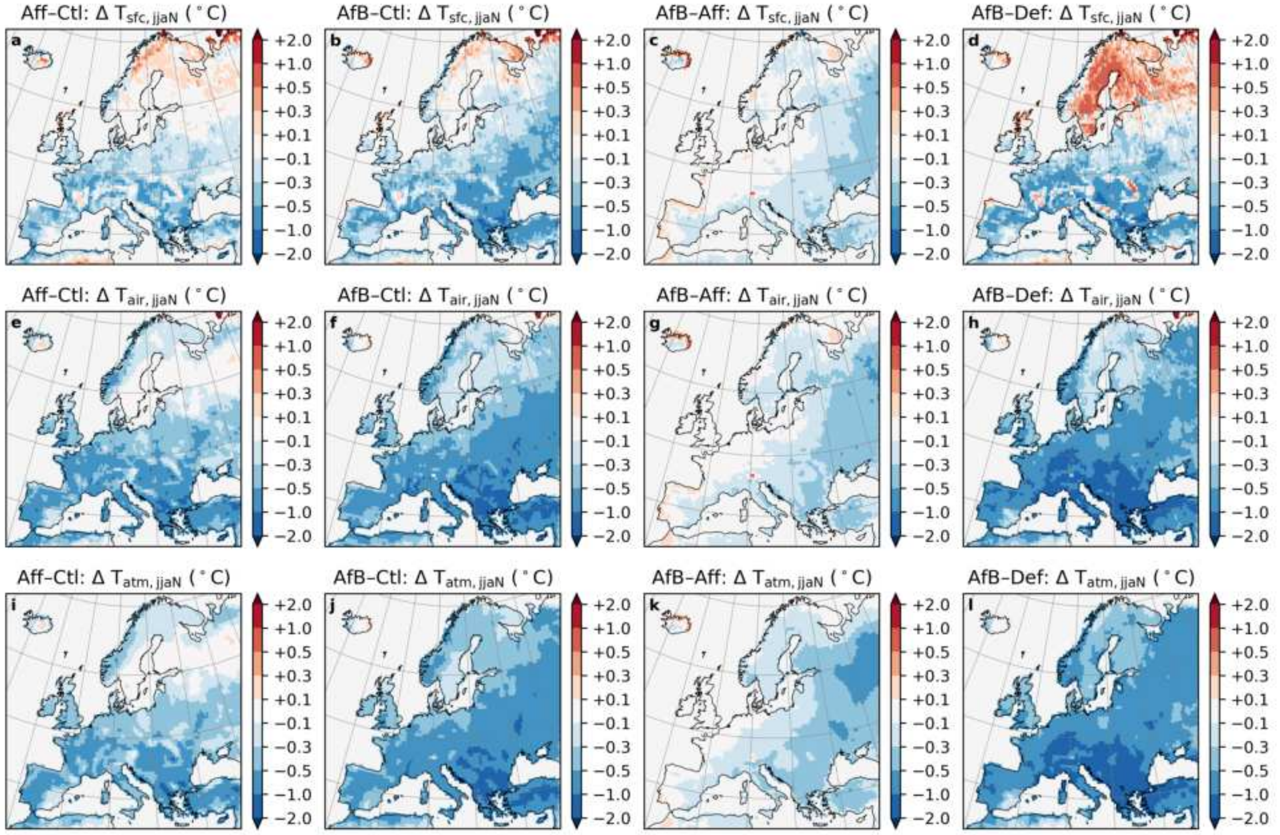

Supplementary Figure S9 | Changes in multi-year (2025-2059, compared to the experiment Ctl) summer (June, July, and August) mean daily minimum land surface temperature ($T_{sfc,jjaN}$: **a-d**), 2-meter air temperature ($T_{air,jjaN}$: **e-h**), and the temperature at the lowest atmospheric level ($T_{atm,jjaN}$: **i-l**) under the forestation scenario (Aff-Ctl: **a,e,i**), under the combining forestation and conversion from coniferous to broadleaf forests scenario (AfB-Ctl: **b,f,j**), the difference between the two scenarios (AfB-Aff: **c,g,k**), and the difference between AfB and the deforestation scenario (AfB-Def: **d,h,l**).

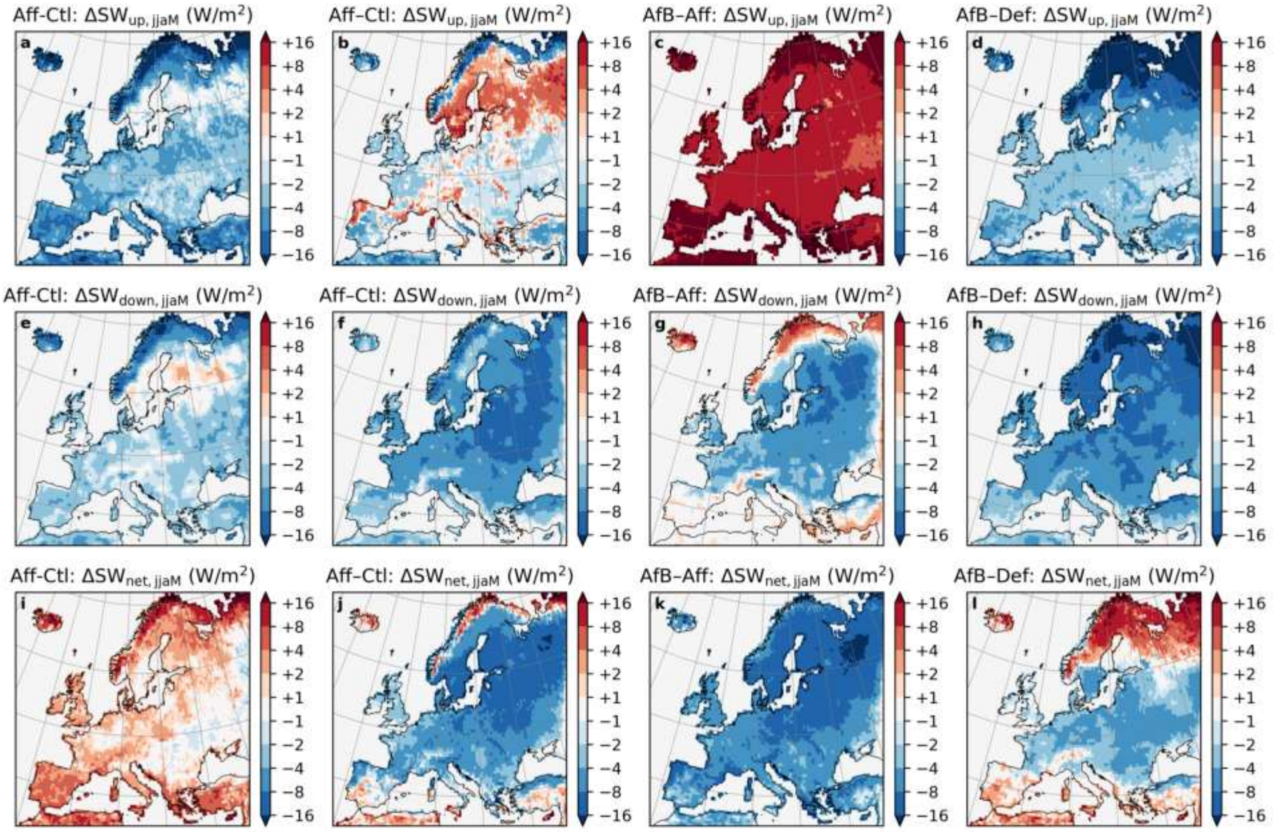

Supplementary Figure S10 | Changes in multi-year (2025-2059, compared to the experiment Ctl) summer (June, July, and August) mean up-welling shortwave radiation ($SW_{up,jjaM}$: **a-d**), down-welling shortwave radiation ($SW_{down,jjaM}$: **e-h**), and net shortwave radiation ($SW_{net,jjaM}$: **i-l**) under the forestation scenario (Aff-Ctl: **a,e,i**), under the combining forestation and conversion from coniferous to broadleaf forests scenario (AfB-Ctl: **b,f,j**), the difference between the two scenarios (AfB-Aff: **c,g,k**), and the difference between AfB and the deforestation scenario (AfB-Def: **d,h,l**).

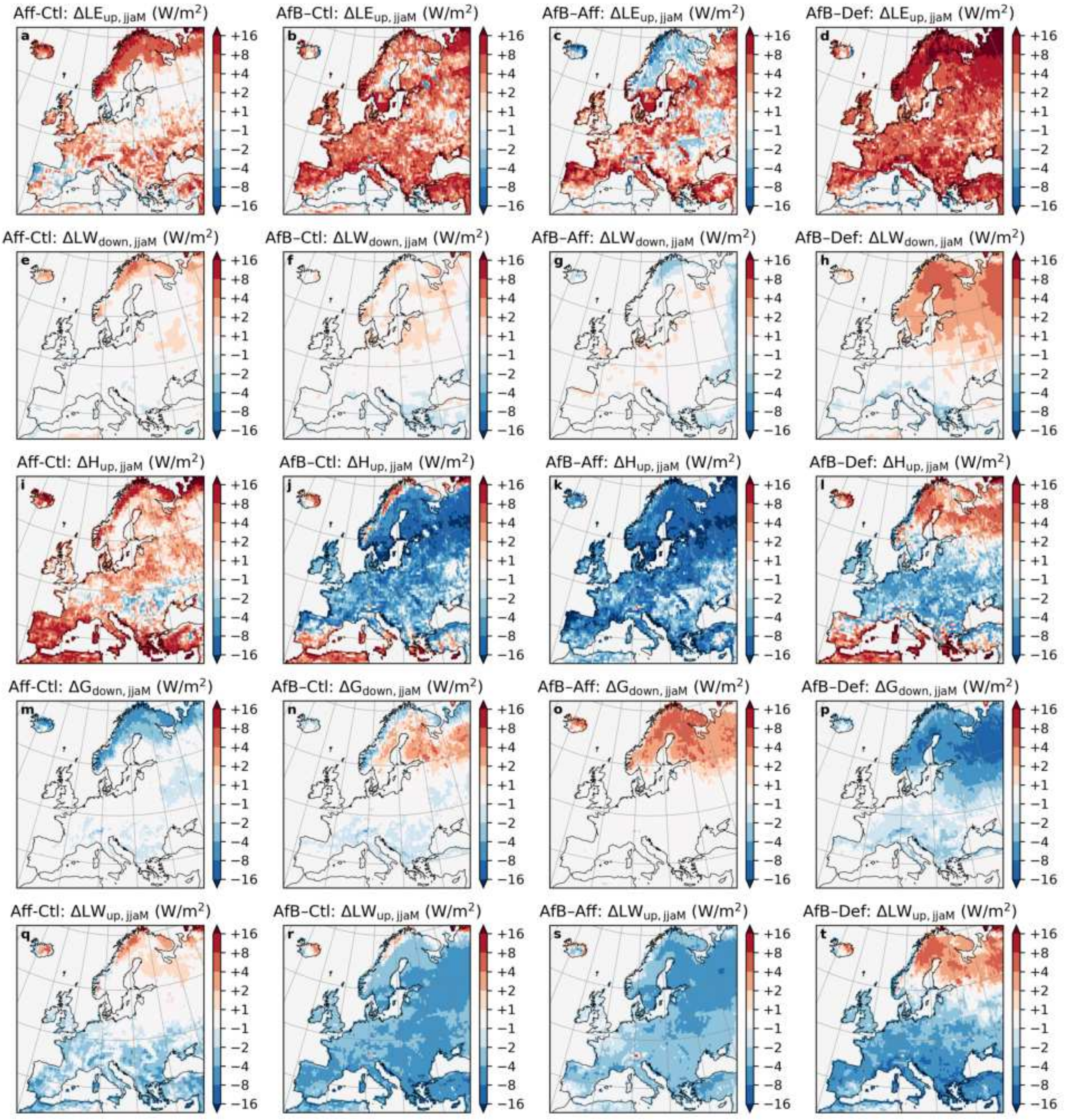

Supplementary Figure S11 | Changes in multi-year (2025-2059, compared to the experiment Ctl) summer (June, July, and August) mean latent heat flux from the land to the atmosphere ($LE_{up,jjaM}$: **a-d**), down-welling longwave radiation ($LW_{down,jjaM}$: **e-h**), sensible heat flux from the land to the atmosphere ($H_{up,jjaM}$: **i-l**), the ground flux (from the land surface to the ground, $G_{down,jjaM}$: **m-p**), and up-welling longwave radiation ($LW_{up,jjaM}$: **q-t**). Changes in these fluxes under the forestation scenario (Aff-Ctl: **a,e,i,m,q**), by the combining scenario of forestation and the conversion from coniferous to broadleaf forests (AfB-Ctl: **b,f,j,n,r**), the difference between the two scenarios (AfB-Aff: **c,g,k,o,s**), and the difference between AfB and the deforestation scenario (AfB-Def: **d,h,l,p,t**).

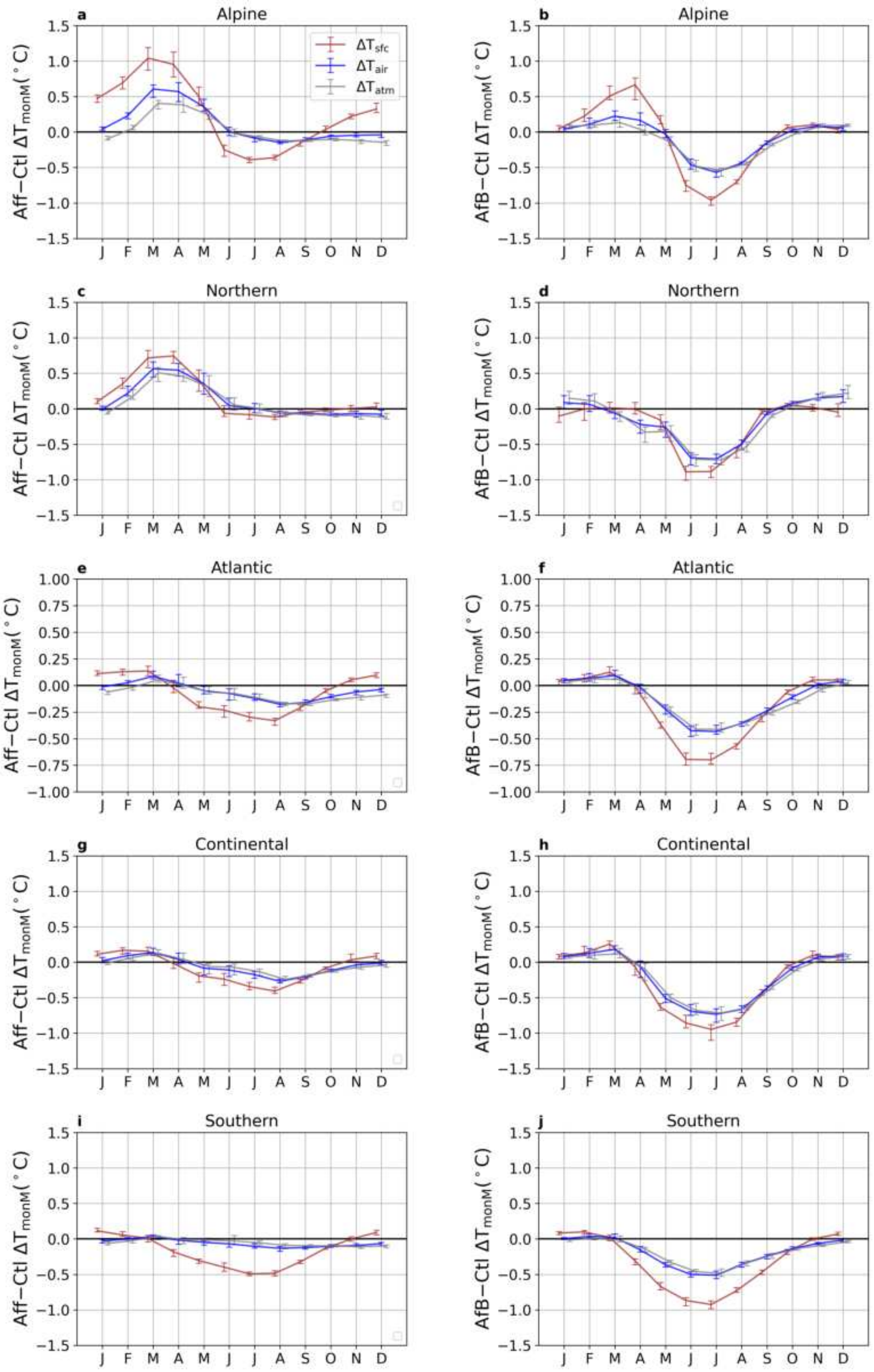

Supplementary Figure S12 | Changes in multi-year mean (2025-2059) monthly mean daily mean temperature (T_{monM}) induced by the forestation scenario (Aff) (**left column**) and the combining scenario of forestation and the conversion from coniferous to broadleaf forests (AfB) (**right column**). The values shown are the regionally averaged temperature change (minus the outputs from the Ctl simulation) over five regions: Alpine (**a-b**), Northern (**c-d**), Atlantic (**e-f**), Continental (**g-h**), and Southern (**i-j**).

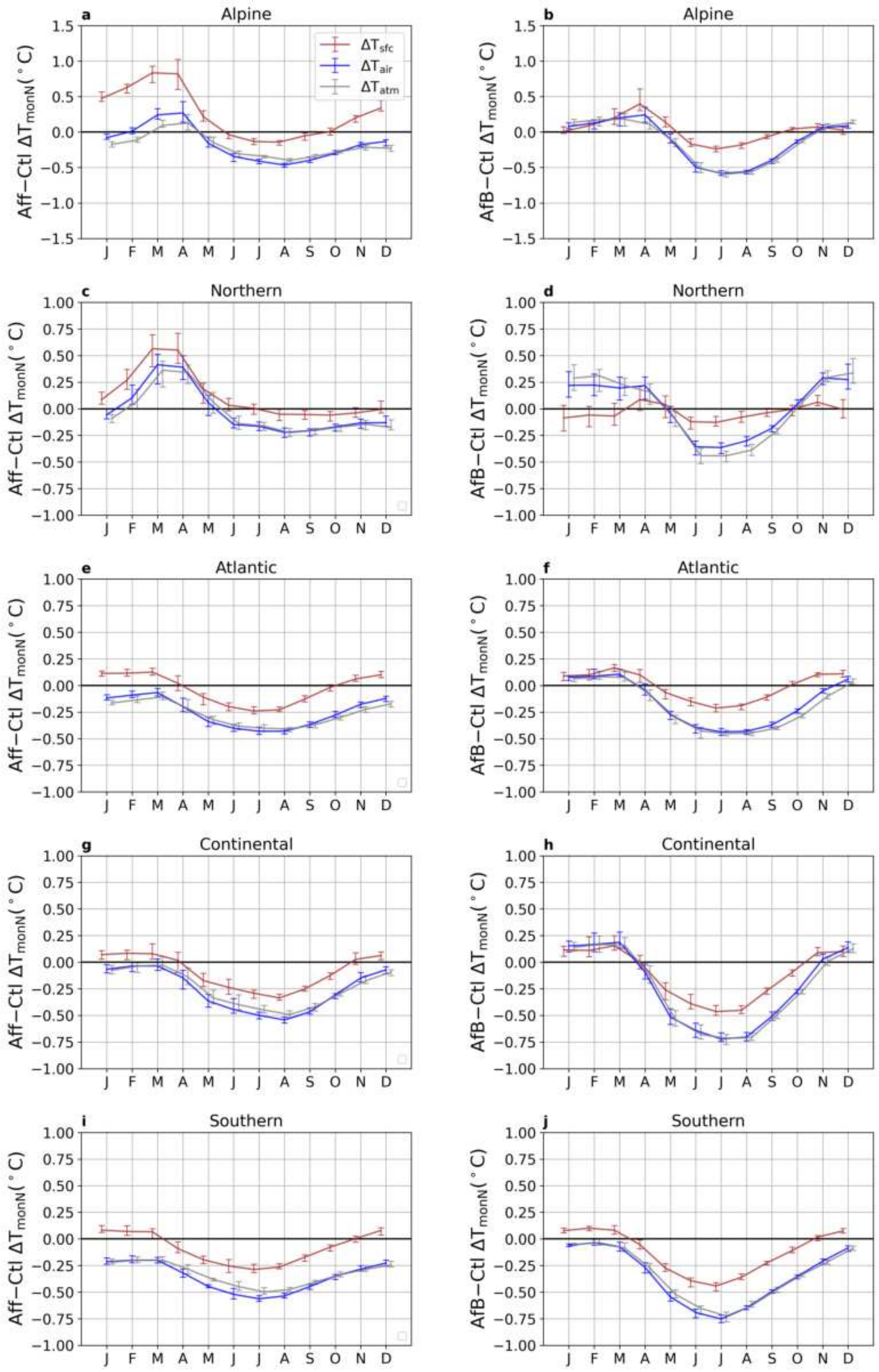

Supplementary Figure S13 | Changes in multi-year mean (2025-2059) monthly mean daily minimum temperature (T_{monN}) induced by the forestation scenario (Aff) (**left column**) and the combining scenario of forestation and the conversion from coniferous to broadleaf forests (AfB) (**right column**). The values shown are the regionally averaged temperature change (minus the outputs from the Ctl simulation) over five regions: Alpine (**a-b**), Northern (**c-d**), Atlantic (**e-f**), Continental (**g-h**), and Southern (**i-j**).

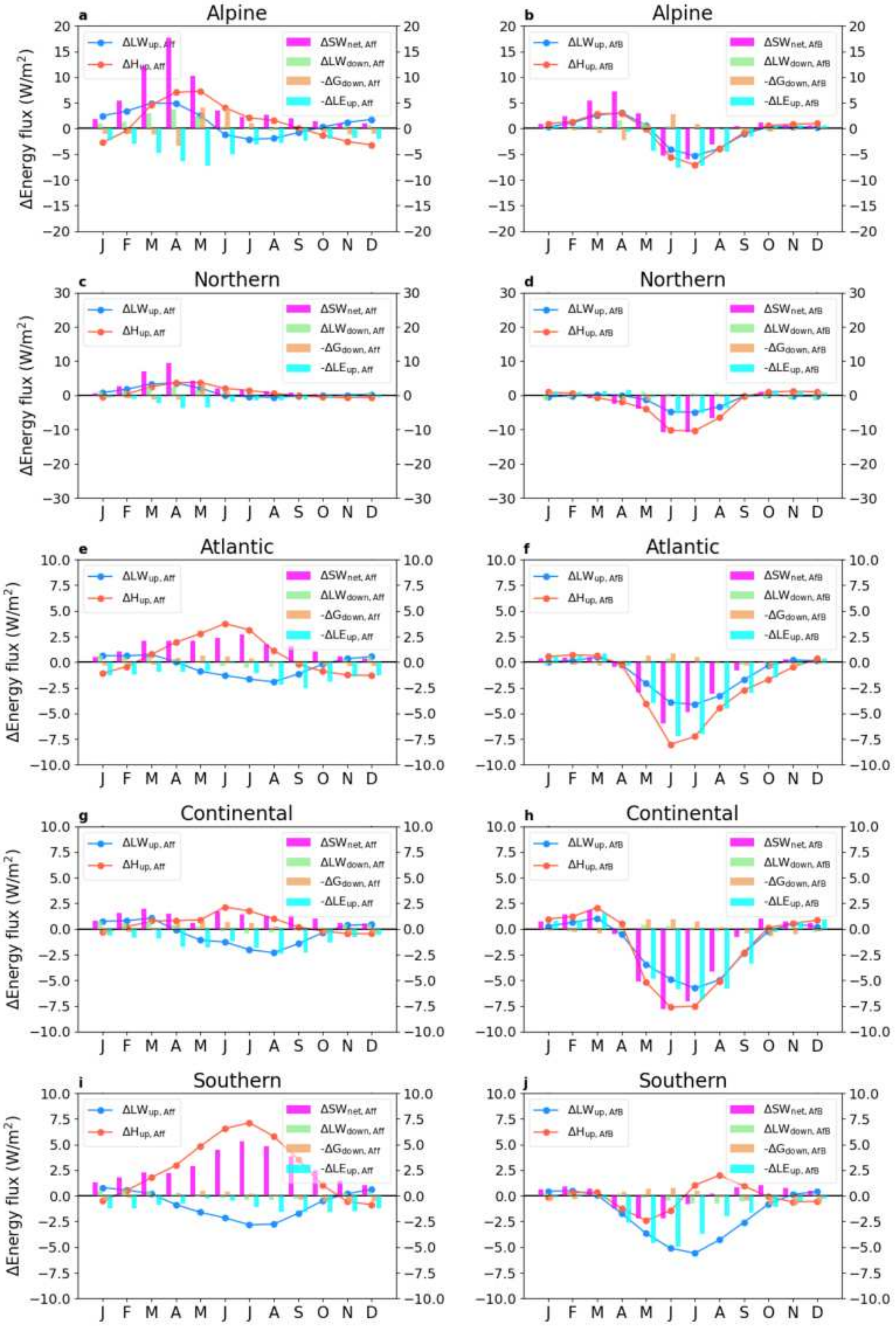

Supplementary Figure S14 | Changes in multi-year mean (2025-2059) monthly mean daily mean up-welling longwave radiation (LW_{up}), sensible heat flux from the land to the atmosphere (H_{up}), net shortwave radiation (SW_{net}), down-welling longwave radiation (LW_{down}), ground energy flux from the land surface to the ground (G_{down}), and latent heat flux from the land to the atmosphere (LE_{up}), induced by the forestation scenario (Aff) (**left column**) and the combining scenario of forestation and the conversion from coniferous to broadleaf forests (AfB) (**right column**). The values shown are the regionally averaged temperature change (minus the outputs from the Ctl simulation) over five regions: Alpine (**a-b**), Northern (**c-d**), Atlantic (**e-f**), Continental (**g-h**), and Southern (**i-j**).

2 Supplementary Tables referred to in the manuscript

Table S1: Average energy fluxes and temperatures in summer over the Alpine region

Energy fluxes (W/m^2)	$SW_{down,jjaM}$	$SW_{up,jjaM}$	$LW_{up,jjaM}$	$LW_{down,jjaM}$
Ctl	184.49±2.04	25.76±0.32	394.45±1.12	335.26±1.32
Brd	183.15±2.08	28.15±0.36	392.95±1.10	335.35±1.35
Def	186.77±2.07	30.52±0.42	395.31±1.12	335.13±1.35
Aff	181.32±2.13	19.78±0.21	392.71±1.10	335.14±1.33
AfB	179.75±2.04	25.82±0.28	390.02±1.07	335.29±1.34
Energy fluxes (W/m^2)	$LE_{up,jjaM}$	$H_{up,jjaM}$	$G_{down,jjaM}$	
Ctl	55.91±0.68	33.73±0.58	8.38±0.44	
Brd	57.50±0.70	29.51±0.61	8.89±0.48	
Def	52.48±0.61	31.67±0.60	10.33±0.57	
Aff	59.55±0.74	36.31±0.69	6.66±0.29	
AfB	62.36±0.82	28.23±0.66	7.18±0.35	
Temperature (°C)	$T_{sfc,jjaX}$	$T_{air,jjaX}$	$T_{atm,jjaX}$	
Ctl	21.71±0.24	18.85±0.22	18.14±0.22	
Brd	21.22±0.23	18.45±0.22	17.78±0.22	
Def	22.37±0.25	18.50±0.22	17.81±0.22	
Aff	20.96±0.23	19.14±0.22	18.39±0.22	
AfB	20.07±0.22	18.47±0.22	17.77±0.21	
Temperature (°C)	$T_{sfc,jjaN}$	$T_{air,jjaN}$	$T_{atm,jjaN}$	
Ctl	9.51±0.20	10.75±0.20	11.53±0.21	
Brd	9.44±0.20	10.64±0.20	11.38±0.20	
Def	9.38±0.20	10.91±0.20	11.68±0.21	
Aff	9.41±0.20	10.34±0.20	11.18±0.21	
AfB	9.32±0.20	10.20±0.20	10.97±0.20	
Temperature (°C)	$T_{sfc,jjaM}$	$T_{air,jjaM}$	$T_{atm,jjaM}$	
Ctl	15.42±0.21	14.90±0.21	14.91±0.21	
Brd	15.15±0.20	14.64±0.20	14.64±0.20	
Def	15.56±0.21	14.82±0.20	14.83±0.21	
Aff	15.09±0.20	14.83±0.20	14.84±0.21	
AfB	14.61±0.20	14.41±0.20	14.41±0.20	

Table S2: Average energy fluxes and temperatures in summer over the Northern region

Energy fluxes (W/m^2)	$SW_{down,jjaM}$	$SW_{up,jjaM}$	$LW_{up,jjaM}$	$LW_{down,jjaM}$
Ctl	170.81±2.81	21.15±0.32	397.43±1.49	344.30±1.54
Brd	167.01±2.75	24.69±0.37	394.63±1.45	344.68±1.49
Def	173.03±2.71	27.55±0.41	396.65±1.47	343.62±1.53
Aff	169.05±2.85	17.74±0.27	397.06±1.51	344.49±1.53
AfB	163.52±2.84	23.29±0.37	393.05±1.41	345.02±1.51
Energy fluxes (W/m^2)	$LE_{up,jjaM}$	$H_{up,jjaM}$	$G_{down,jjaM}$	
Ctl	60.26±0.95	27.02±0.66	4.65±0.39	
Brd	62.71±1.09	19.64±0.48	5.54±0.45	
Def	57.72±0.99	22.58±0.58	7.49±0.62	
Aff	61.85±0.93	28.36±0.76	3.96±0.34	
AfB	64.98±1.13	17.98±0.52	4.84±0.41	
Temperature (°C)	$T_{sfc,jjaX}$	$T_{air,jjaX}$	$T_{atm,jjaX}$	
Ctl	21.36±0.32	19.63±0.30	19.07±0.30	
Brd	20.41±0.30	18.85±0.29	18.33±0.29	
Def	21.56±0.31	19.01±0.29	18.47±0.29	
Aff	21.15±0.32	19.82±0.31	19.23±0.31	
AfB	19.78±0.29	18.72±0.28	18.19±0.28	
Temperature (°C)	$T_{sfc,jjaN}$	$T_{air,jjaN}$	$T_{atm,jjaN}$	
Ctl	10.39±0.25	11.40±0.25	11.97±0.25	
Brd	10.30±0.25	11.25±0.25	11.77±0.25	
Def	10.11±0.25	11.47±0.25	12.07±0.25	
Aff	10.38±0.25	11.22±0.25	11.79±0.26	
AfB	10.27±0.25	11.05±0.25	11.54±0.25	
Temperature (°C)	$T_{sfc,jjaM}$	$T_{air,jjaM}$	$T_{atm,jjaM}$	
Ctl	16.00±0.27	15.76±0.27	15.73±0.27	
Brd	15.48±0.26	15.29±0.26	15.25±0.27	
Def	15.87±0.27	15.49±0.26	15.47±0.27	
Aff	15.92±0.27	15.77±0.27	15.73±0.28	
AfB	15.19±0.26	15.12±0.26	15.06±0.26	

Table S3: Average energy fluxes and temperatures in summer over the Atlantic region

Energy fluxes (W/m^2)	$SW_{down,jjaM}$	$SW_{up,jjaM}$	$LW_{up,jjaM}$	$LW_{down,jjaM}$
Ctl	185.58±2.01	25.82±0.29	408.91±1.50	353.91±1.31
Brd	184.71±1.92	28.40±0.29	407.87±1.49	354.17±1.35
Def	187.32±1.91	29.99±0.30	410.27±1.52	354.26±1.35
Aff	183.30±2.07	21.25±0.24	407.29±1.47	353.45±1.32
AfB	181.47±1.92	26.34±0.26	405.14±1.44	353.97±1.33
Energy fluxes (W/m^2)	$LE_{up,jjaM}$	$H_{up,jjaM}$	$G_{down,jjaM}$	
Ctl	59.87±0.97	38.69±0.69	5.12±0.18	
Brd	62.63±1.14	33.85±0.82	5.04±0.17	
Def	59.22±1.08	35.59±0.75	5.35±0.17	
Aff	60.99±0.95	41.37±0.69	4.79±0.17	
AfB	66.14±1.19	32.12±0.89	4.65±0.16	
Temperature (°C)	$T_{sfc,jjaX}$	$T_{air,jjaX}$	$T_{atm,jjaX}$	
Ctl	24.56±0.33	21.67±0.29	20.88±0.29	
Brd	24.14±0.33	21.38±0.29	20.63±0.29	
Def	25.23±0.34	21.46±0.29	20.67±0.29	
Aff	24.03±0.32	21.90±0.29	21.09±0.29	
AfB	23.14±0.31	21.31±0.29	20.57±0.28	
Temperature (°C)	$T_{sfc,jjaN}$	$T_{air,jjaN}$	$T_{atm,jjaN}$	
Ctl	12.48±0.22	13.47±0.22	14.01±0.22	
Brd	12.48±0.22	13.45±0.22	13.98±0.22	
Def	12.50±0.22	13.70±0.22	14.25±0.23	
Aff	12.26±0.22	13.05±0.22	13.61±0.22	
AfB	12.29±0.22	13.04±0.22	13.56±0.22	
Temperature (°C)	$T_{sfc,jjaM}$	$T_{air,jjaM}$	$T_{atm,jjaM}$	
Ctl	18.06±0.26	17.52±0.25	17.40±0.26	
Brd	17.88±0.26	17.37±0.25	17.26±0.25	
Def	18.29±0.26	17.54±0.25	17.43±0.26	
Aff	17.77±0.26	17.40±0.25	17.28±0.25	
AfB	17.40±0.25	17.11±0.25	17.01±0.25	

Table S4: Average energy fluxes and temperatures in summer over the Continental region

Energy fluxes (W/m^2)	$SW_{down,jjaM}$	$SW_{up,jjaM}$	$LW_{up,jjaM}$	$LW_{down,jjaM}$
Ctl	210.45±2.67	29.78±0.36	421.03±1.60	354.92±1.52
Brd	207.83±2.74	31.64±0.40	418.99±1.50	355.02±1.52
Def	211.54±2.71	33.47±0.42	421.94±1.59	354.93±1.53
Aff	208.65±2.84	26.51±0.33	419.17±1.60	354.58±1.52
AfB	204.01±2.70	29.68±0.37	415.83±1.48	354.89±1.51
Energy fluxes (W/m^2)	$LE_{up,jjaM}$	$H_{up,jjaM}$	$G_{down,jjaM}$	
Ctl	70.78±0.80	36.63±1.04	6.81±0.21	
Brd	73.75±0.84	31.44±0.94	6.71±0.21	
Def	69.32±0.79	34.20±0.96	7.21±0.22	
Aff	72.59±0.84	38.27±1.14	6.32±0.20	
AfB	76.97±1.01	29.88±0.97	6.18±0.20	
Temperature (°C)	$T_{sfc,jjaX}$	$T_{air,jjaX}$	$T_{atm,jjaX}$	
Ctl	27.48±0.37	24.48±0.31	23.73±0.30	
Brd	26.79±0.34	23.95±0.29	23.24±0.28	
Def	28.02±0.37	24.14±0.30	23.39±0.29	
Aff	26.99±0.37	24.70±0.32	23.92±0.31	
AfB	25.84±0.33	23.81±0.29	23.11±0.29	
Temperature (°C)	$T_{sfc,jjaN}$	$T_{air,jjaN}$	$T_{atm,jjaN}$	
Ctl	13.80±0.24	15.03±0.24	15.77±0.25	
Brd	13.70±0.24	14.90±0.23	15.60±0.24	
Def	13.76±0.24	15.26±0.24	16.01±0.24	
Aff	13.51±0.24	14.55±0.24	15.33±0.25	
AfB	13.37±0.24	14.35±0.24	15.08±0.24	
Temperature (°C)	$T_{sfc,jjaM}$	$T_{air,jjaM}$	$T_{atm,jjaM}$	
Ctl	20.21±0.27	19.78±0.26	19.82±0.26	
Brd	19.86±0.26	19.46±0.25	19.49±0.25	
Def	20.36±0.27	19.75±0.26	19.77±0.26	
Aff	19.88±0.28	19.59±0.27	19.66±0.27	
AfB	19.31±0.26	19.08±0.25	19.13±0.25	

Table S5: Average energy fluxes and temperatures in summer over the Southern region

Energy fluxes (W/m^2)	$SW_{down,jjaM}$	$SW_{up,jjaM}$	$LW_{up,jjaM}$	$LW_{down,jjaM}$
Ctl	268.68±1.65	44.84±0.33	458.60±1.68	361.39±1.55
Brd	268.07±1.70	46.47±0.35	457.49±1.70	361.41±1.54
Def	269.19±1.68	47.93±0.37	459.77±1.70	361.78±1.57
Aff	266.42±1.60	37.71±0.24	456.02±1.67	361.06±1.55
AfB	265.85±1.71	42.93±0.29	453.60±1.71	360.74±1.52
Energy fluxes (W/m^2)	$LE_{up,jjaM}$	$H_{up,jjaM}$	$G_{down,jjaM}$	
Ctl	53.00±1.10	64.76±1.11	6.56±0.16	
Brd	54.50±1.16	62.26±1.16	6.46±0.15	
Def	52.22±1.08	61.95±1.07	6.71±0.17	
Aff	54.06±1.16	71.25±1.24	6.37±0.15	
AfB	56.56±1.42	65.29±1.43	6.17±0.15	
Temperature (°C)	$T_{sfc,jjaX}$	$T_{air,jjaX}$	$T_{atm,jjaX}$	
Ctl	37.33±0.37	30.95±0.31	29.80±0.30	
Brd	37.00±0.38	30.67±0.31	29.54±0.30	
Def	37.94±0.38	30.65±0.30	29.49±0.30	
Aff	36.38±0.36	31.45±0.31	30.25±0.30	
AfB	35.66±0.37	30.87±0.32	29.72±0.31	
Temperature (°C)	$T_{sfc,jjaN}$	$T_{air,jjaN}$	$T_{atm,jjaN}$	
Ctl	18.09±0.23	19.63±0.23	20.44±0.24	
Brd	18.02±0.23	19.54±0.23	20.33±0.24	
Def	18.08±0.23	19.82±0.23	20.63±0.23	
Aff	17.83±0.23	19.10±0.23	19.98±0.24	
AfB	17.69±0.23	18.93±0.23	19.77±0.24	
Temperature (°C)	$T_{sfc,jjaM}$	$T_{air,jjaM}$	$T_{atm,jjaM}$	
Ctl	26.51±0.27	25.09±0.26	25.00±0.26	
Brd	26.33±0.28	24.90±0.26	24.80±0.26	
Def	26.71±0.28	25.06±0.26	24.95±0.26	
Aff	26.06±0.27	24.99±0.26	24.94±0.26	
AfB	25.67±0.28	24.63±0.27	24.57±0.27	

3 Supplementary Figures of other temperatures

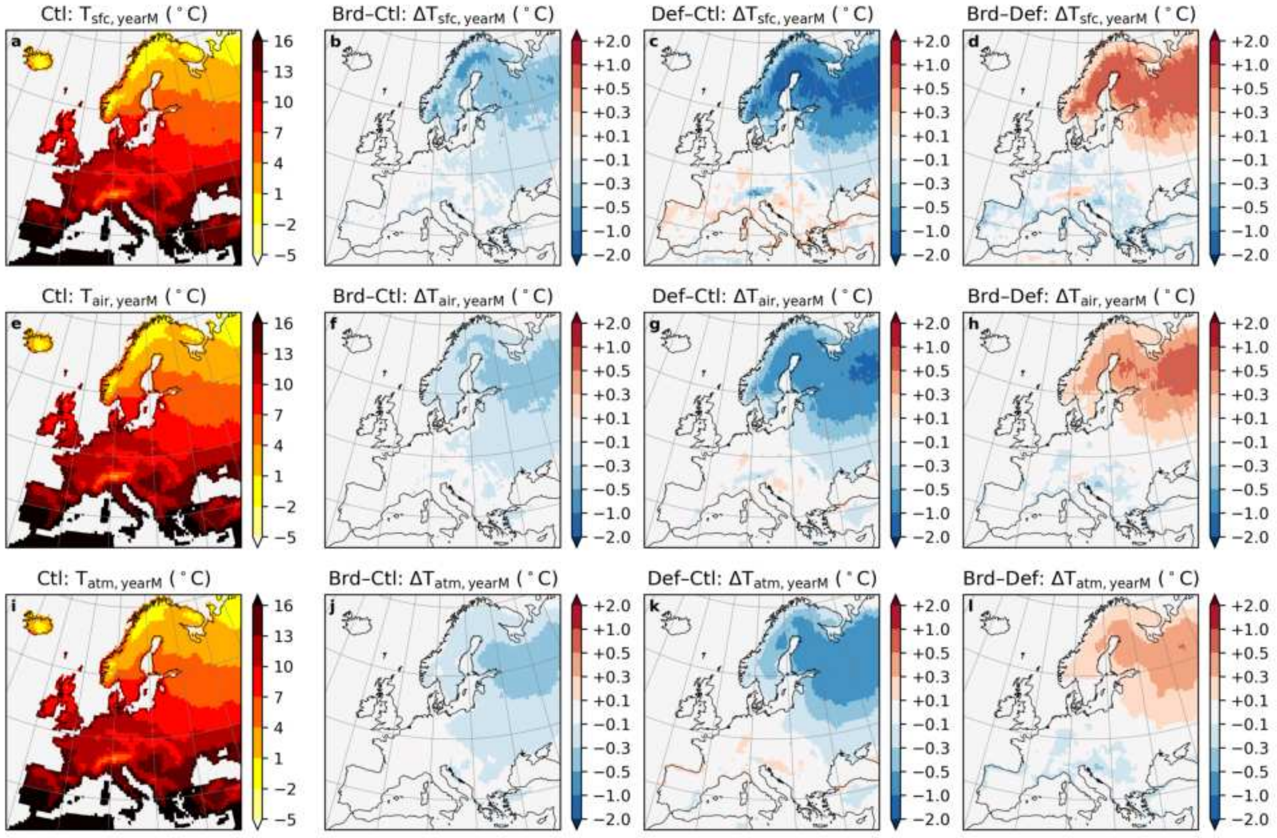

Supplementary Figure S15 | Multi-year (2025-2059) annual mean daily mean land surface temperature ($T_{sfc,yearM}$: **a**), 2-meter air temperature ($T_{air,yearM}$: **e**), and the temperature at the lowest atmospheric level ($T_{atm,yearM}$: **i**), under the present-day forest scenario (Ctl). Changes in these temperatures under the conversion from coniferous to broadleaf forests scenario (Brd-Ctl: **b,f,j**), by the deforestation scenario (Def-Ctl: **c,g,k**), and the difference between the two scenarios (Brd-Def: **d,h,l**).

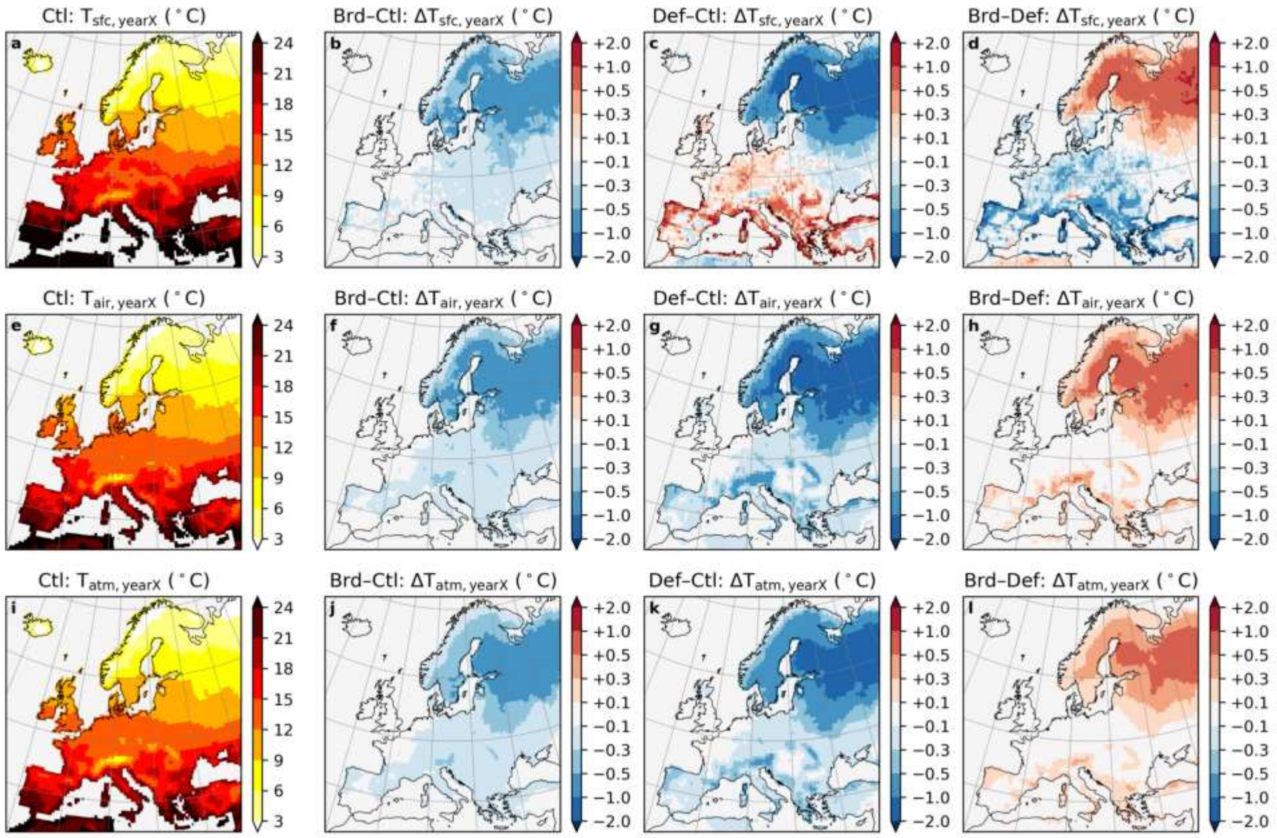

Supplementary Figure S16 | Multi-year (2025-2059) annual mean daily maximum land surface temperature ($T_{sfc,yearX}$: **a**), 2-meter air temperature ($T_{air,yearX}$: **e**), and the temperature at the lowest atmospheric level ($T_{atm,yearX}$: **i**), under the present-day forest scenario (Ctl). Changes in these temperatures under the conversion from coniferous to broadleaf forests scenario (Brd-Ctl: **b,f,j**), by the deforestation scenario (Def-Ctl: **c,g,k**), and the difference between the two scenarios (Brd-Def: **d,h,l**).

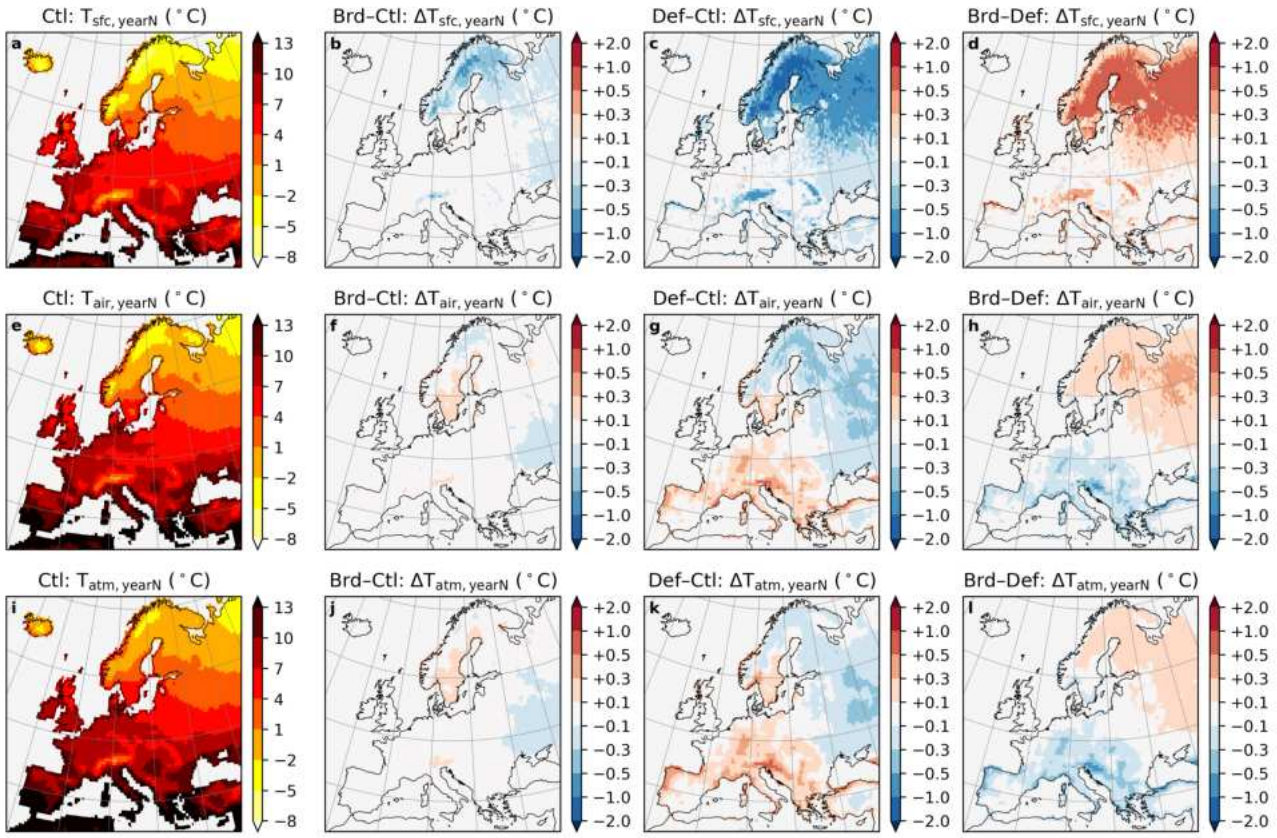

Supplementary Figure S17 | Multi-year (2025-2059) annual mean daily maximum land surface temperature ($T_{sfc,yearN}$: **a**), 2-meter air temperature ($T_{air,yearN}$: **e**), and the temperature at the lowest atmospheric level ($T_{atm,yearN}$: **i**), under the present-day forest scenario (Ctl). Changes in these temperatures under the conversion from coniferous to broadleaf forests scenario (Brd-Ctl: **b,f,j**), by the deforestation scenario (Def-Ctl: **c,g,k**), and the difference between the two scenarios (Brd-Def: **d,h,l**).

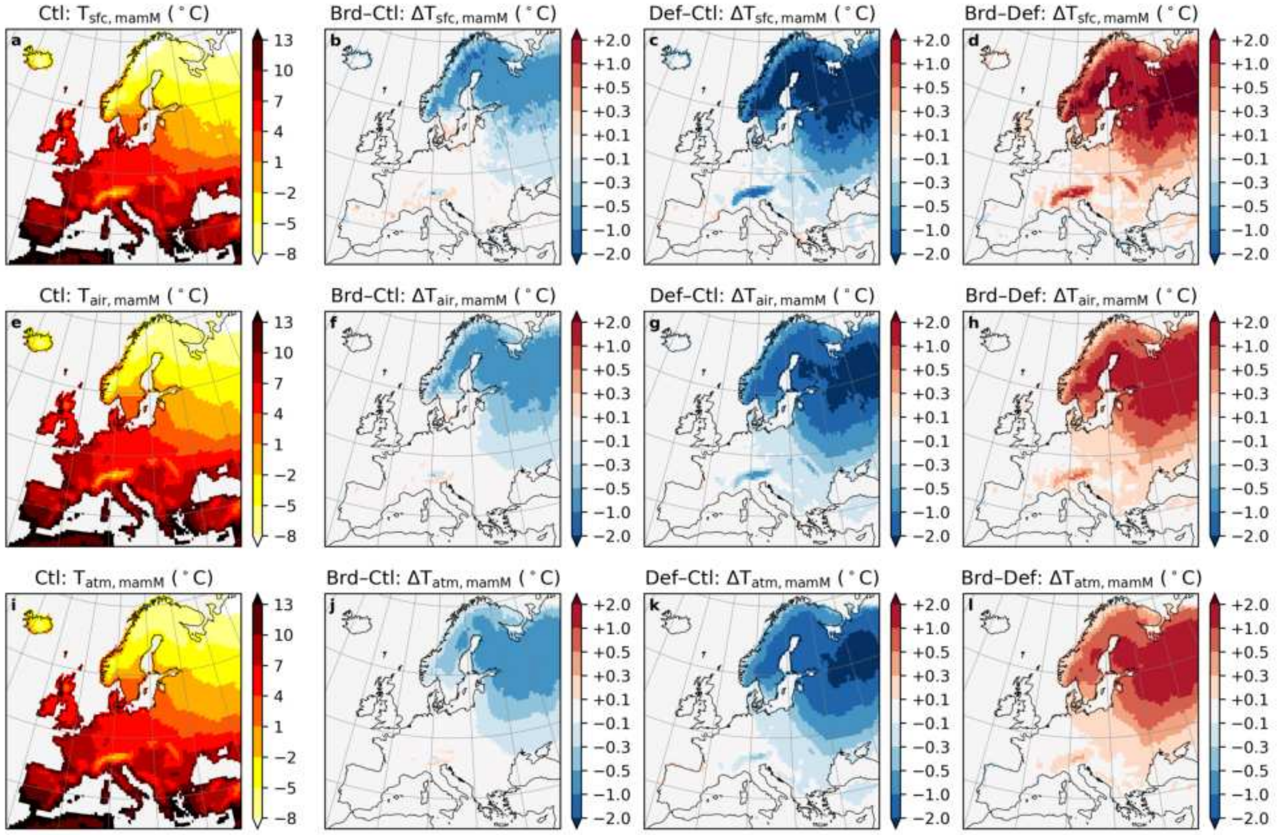

Supplementary Figure S18 | Multi-year (2025-2059) summer (March, April, and May) mean daily mean land surface temperature ($T_{sfc,mamM}$: **a**), 2-meter air temperature ($T_{air,mamM}$: **e**), and the temperature at the lowest atmospheric level ($T_{atm,mamM}$: **i**), under the present-day forest scenario (Ctl). Changes in these temperatures under the conversion from coniferous to broadleaf forests scenario (Brd-Ctl: **b,f,j**), by the deforestation scenario (Def-Ctl: **c,g,k**), and the difference between the two scenarios (Brd-Def: **d,h,l**).

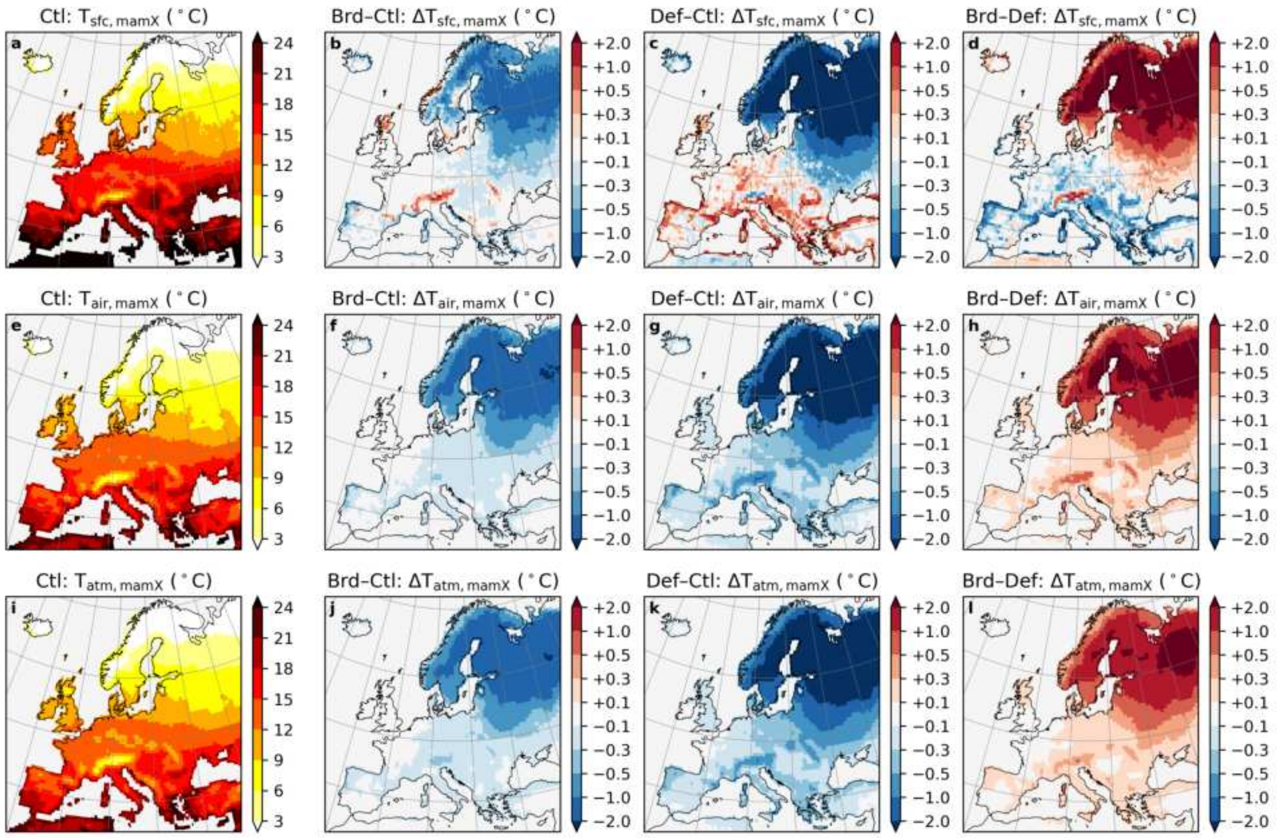

Supplementary Figure S19 | Multi-year (2025-2059) summer (March, April, and May) mean daily maximum land surface temperature ($T_{sfc,mamX}$: **a**), 2-meter air temperature ($T_{air,mamX}$: **e**), and the temperature at the lowest atmospheric level ($T_{atm,mamX}$: **i**), under the present-day forest scenario (Ctl). Changes in these temperatures under the conversion from coniferous to broadleaf forests scenario (Brd-Ctl: **b,f,j**), by the deforestation scenario (Def-Ctl: **c,g,k**), and the difference between the two scenarios (Brd-Def: **d,h,l**).

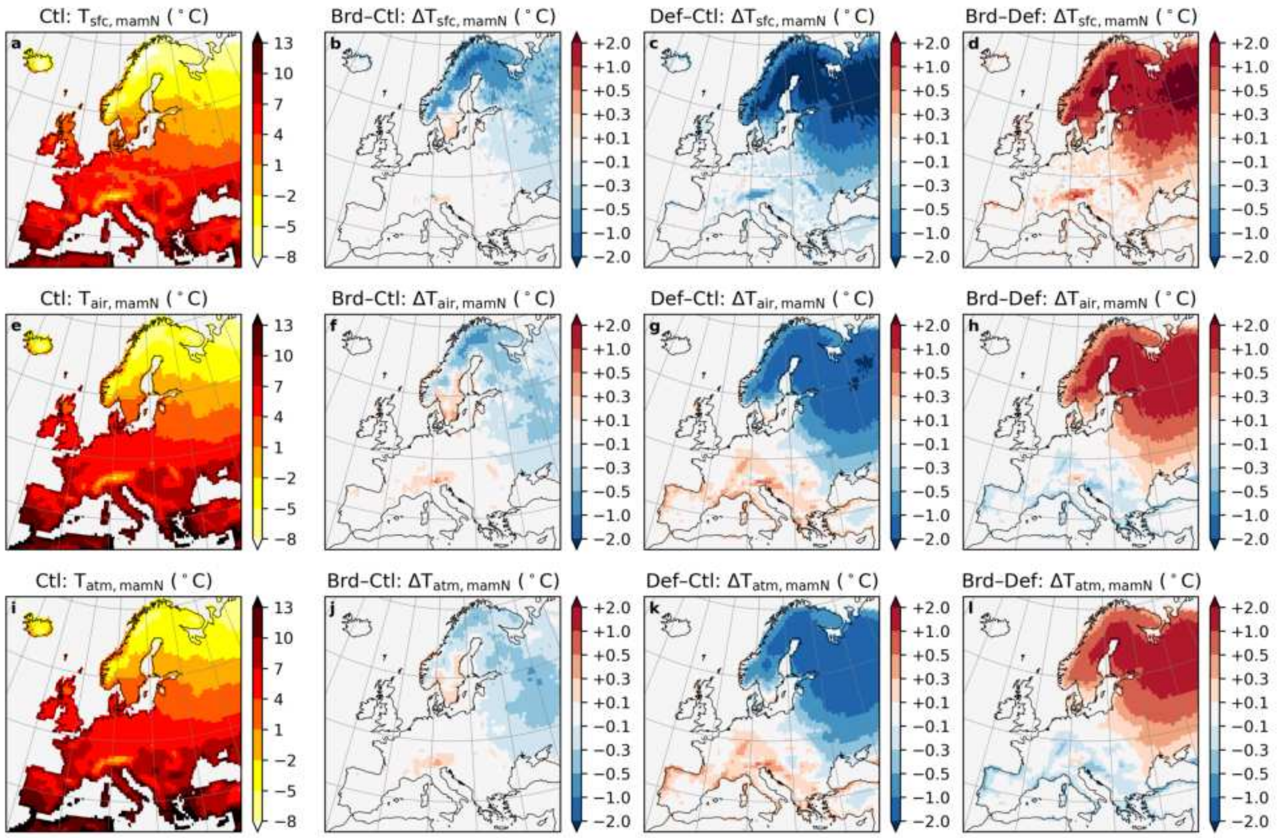

Supplementary Figure S20 | Multi-year (2025-2059, in the experiment Ctl) summer (March, April, and May) mean daily maximum land surface temperature ($T_{sfc,mamN}$: **a**), 2-meter air temperature ($T_{air,mamN}$: **e**), and the temperature at the lowest atmospheric level ($T_{atm,mamN}$: **i**). Changes in these temperatures under the conversion from coniferous to broadleaf forests scenario (Brd-Ctl: **b,f,j**), by the deforestation scenario (Def-Ctl: **c,g,k**), and the difference between the two scenarios (Brd-Def: **d,h,l**).

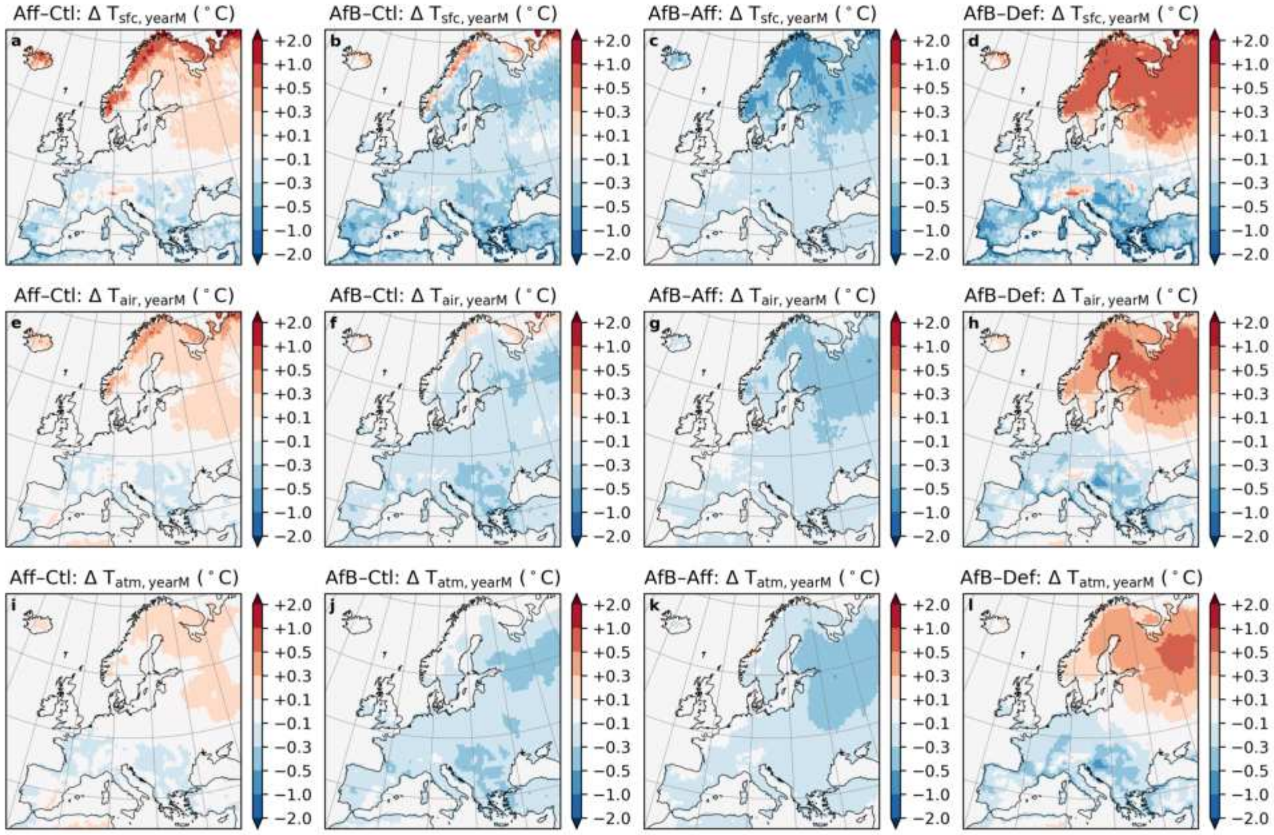

Supplementary Figure S21 | Changes in multi-year (2025-2059, compared to the experiment Ctl) annual mean daily mean land surface temperature ($T_{sfc,yearM}$: **a-d**), 2-meter air temperature ($T_{air,yearM}$: **e-h**), and the temperature at the lowest atmospheric level ($T_{atm,yearM}$: **i-l**) under the forestation scenario (Aff-Ctl: **a,e,i**), by the combining forestation and conversion from coniferous to broadleaf forests scenario (AfB-Ctl: **b,f,j**), the difference between the two scenarios (Brd-Def: **c,g,k**), and the difference between AfB and the deforestation scenario (AfB-Def: **d,h,l**).

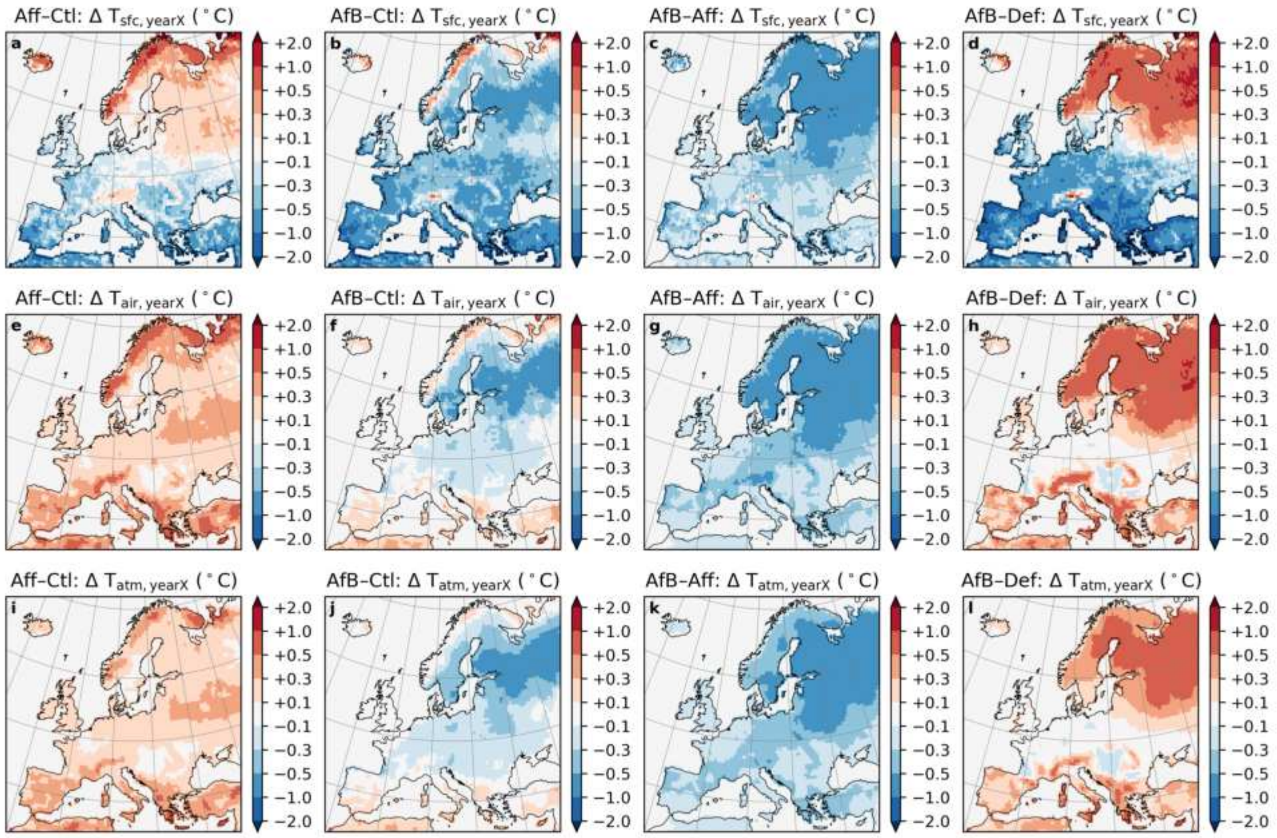

Supplementary Figure S22 | Changes in multi-year (2025-2059, compared to the experiment Ctl) annual mean daily maximum land surface temperature ($T_{\mathrm{sfc,yearX}}$: **a-d**), 2-meter air temperature ($T_{\mathrm{air,yearX}}$: **e-h**), and the temperature at the lowest atmospheric level ($T_{\mathrm{atm,yearX}}$: **i-l**) under the forestation scenario (Aff-Ctl: **a,e,i**), by the combining forestation and conversion from coniferous to broadleaf forests scenario (AfB-Ctl: **b,f,j**), the difference between the two scenarios (Brd-Def: **c,g,k**), and the difference between AfB and the deforestation scenario (AfB-Def: **d,h,l**).

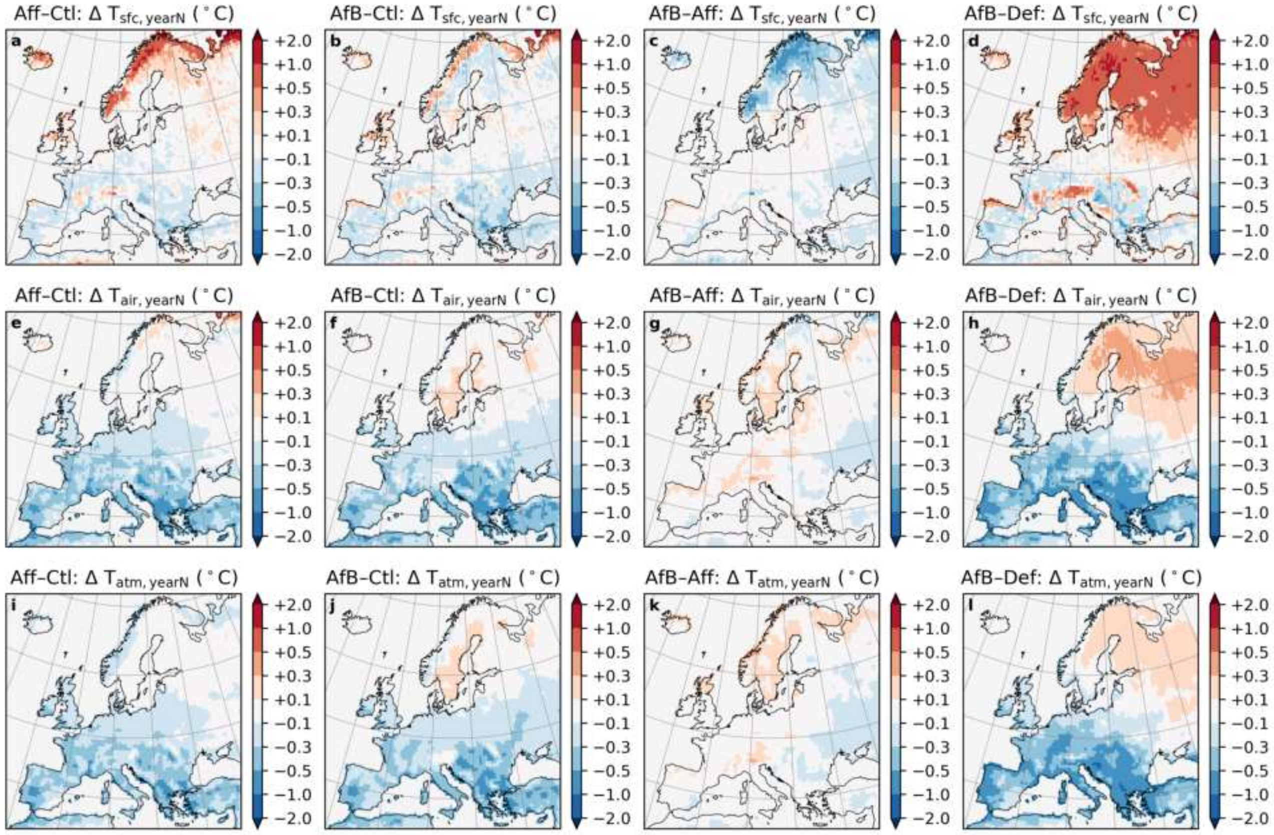

Supplementary Figure S23 | Changes in multi-year (2025-2059, compared to the experiment Ctl) annual mean daily minimum land surface temperature ($T_{sfc,yearN}$: **a-d**), 2-meter air temperature ($T_{air,yearN}$: **e-h**), and the temperature at the lowest atmospheric level ($T_{atm,yearN}$: **i-l**) under the forestation scenario (Aff-Ctl: **a,e,i**), by the combining forestation and conversion from coniferous to broadleaf forests scenario (AfB-Ctl: **b,f,j**), the difference between the two scenarios (Brd-Def: **c,g,k**), and the difference between AfB and the deforestation scenario (AfB-Def: **d,h,l**).

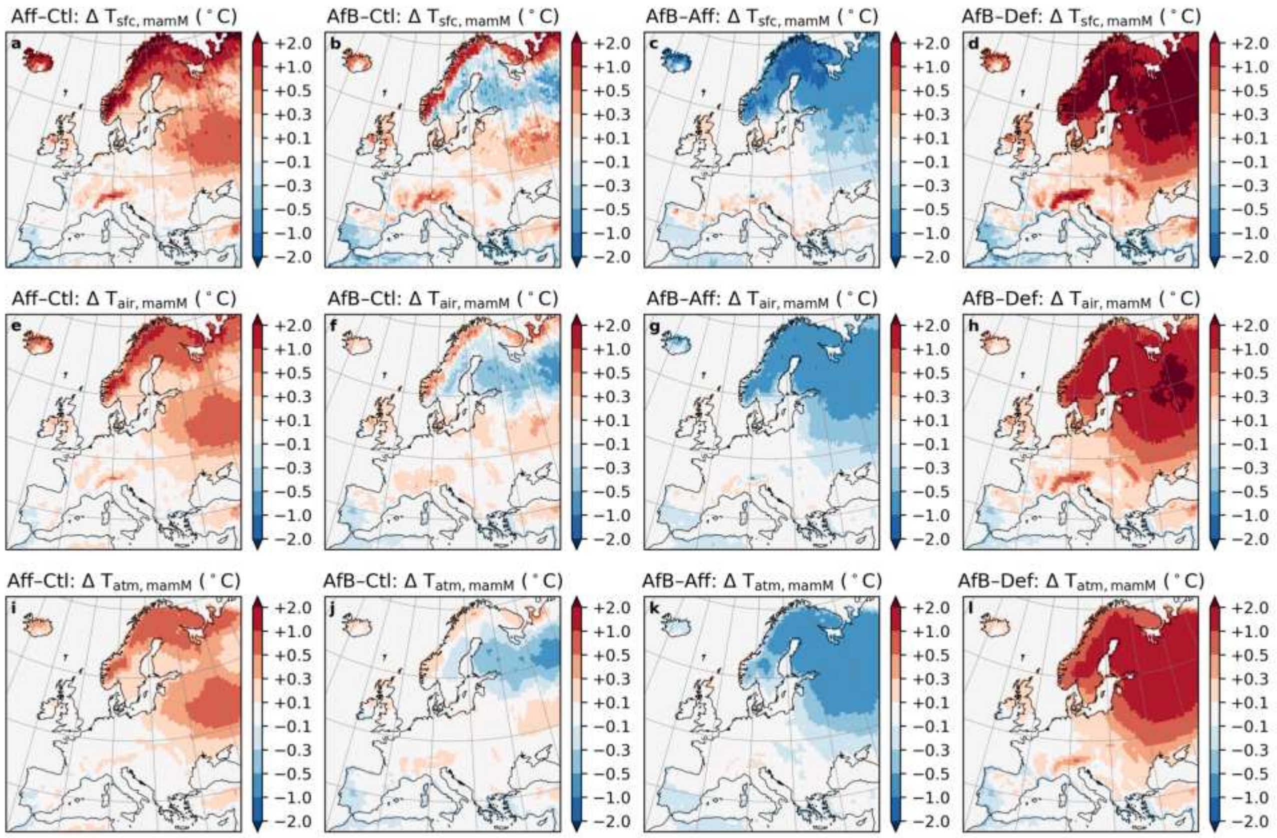

Supplementary Figure S24 | Changes in multi-year (2025-2059, compared to the experiment Ctl) spring (March, April, and May) mean daily mean land surface temperature ($T_{sfc,mamM}$: **a-d**), 2-meter air temperature ($T_{air,mamM}$: **e-h**), and the temperature at the lowest atmospheric level ($T_{atm,mamM}$: **i-l**) under the forestation scenario (Aff-Ctl: **a,e,i**), by the combining forestation and conversion from coniferous to broadleaf forests scenario (AfB-Ctl: **b,f,j**), the difference between the two scenarios (Brd-Def: **c,g,k**), and the difference between AfB and the deforestation scenario (AfB-Def: **d,h,l**).

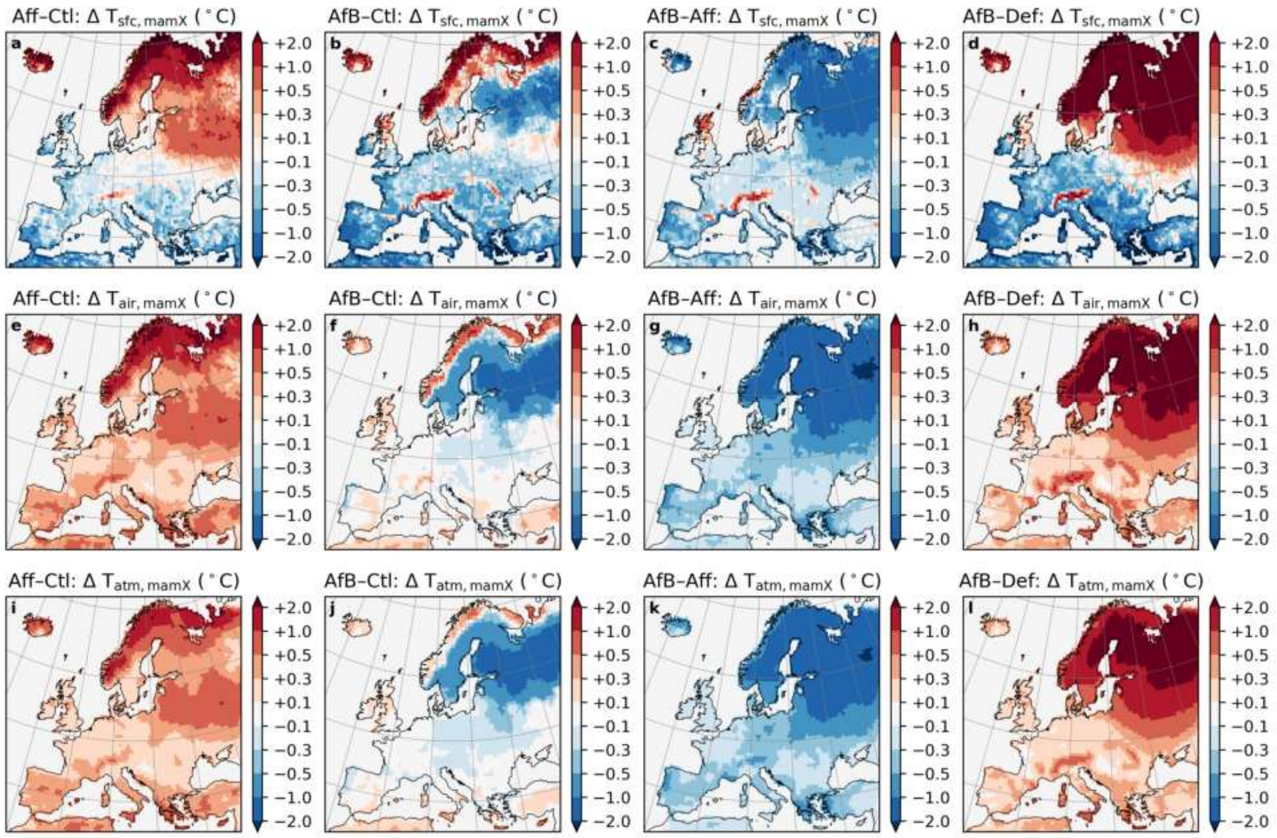

Supplementary Figure S25 | Changes in multi-year (2025-2059, compared to the experiment Ctl) spring (March, April, and May) mean daily maximum land surface temperature ($T_{sfc,mamX}$: **a-d**), 2-meter air temperature ($T_{air,mamX}$: **e-h**), and the temperature at the lowest atmospheric level ($T_{atm,mamX}$: **i-l**) under the forestation scenario (Aff-Ctl: **a,e,i**), by the combining forestation and conversion from coniferous to broadleaf forests scenario (AfB-Ctl: **b,f,j**), the difference between the two scenarios (Brd-Def: **c,g,k**), and the difference between AfB and the deforestation scenario (AfB-Def: **d,h,l**).

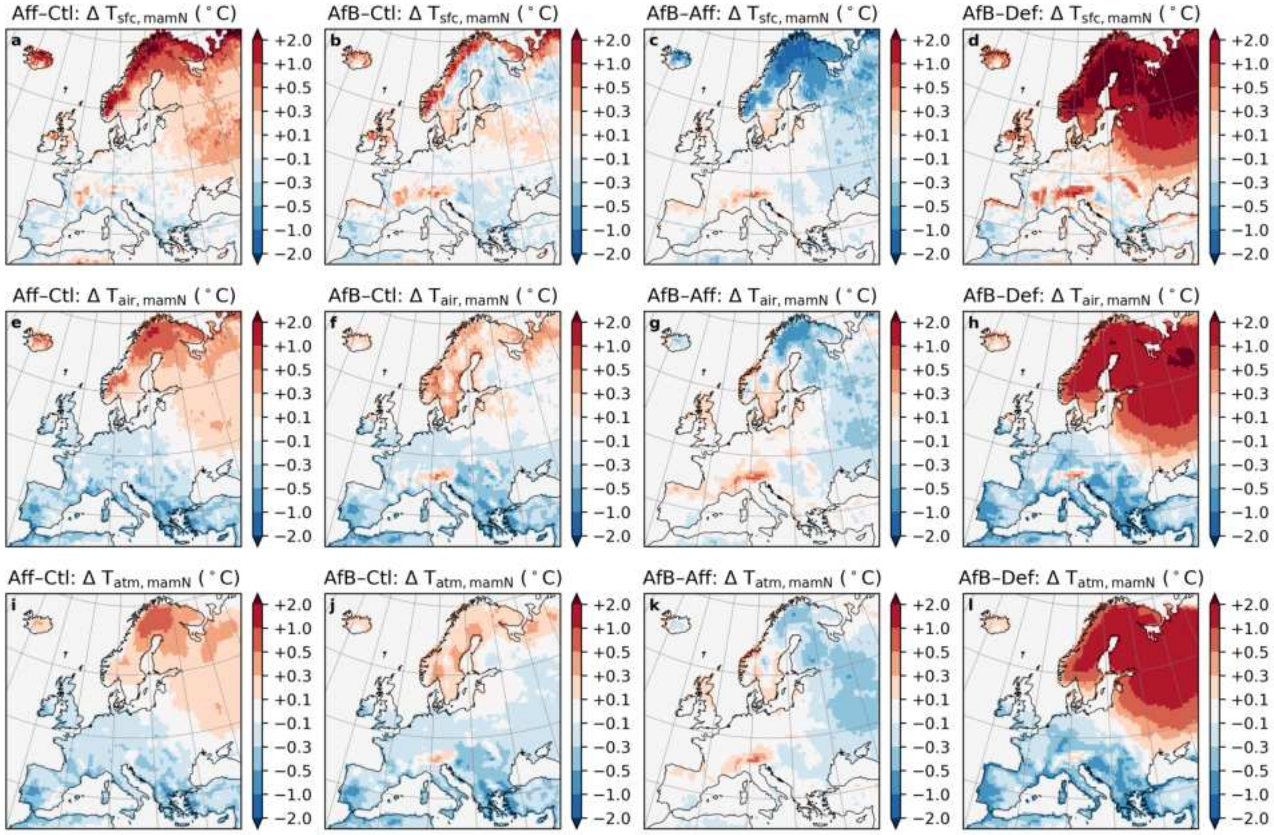

Supplementary Figure S26 | Changes in multi-year (2025-2059, compared to the experiment Ctl) spring (March, April, and May) mean daily minimum land surface temperature ($T_{sfc,mamN}$: **a-d**), 2-meter air temperature ($T_{air,mamN}$: **e-h**), and the temperature at the lowest atmospheric level ($T_{atm,mamN}$: **i-l**) under the forestation scenario (Aff-Ctl: **a,e,i**), by the combining forestation and conversion from coniferous to broadleaf forests scenario (AfB-Ctl: **b,f,j**), the difference between the two scenarios (Brd-Def: **c,g,k**), and the difference between AfB and the deforestation scenario (AfB-Def: **d,h,l**).

4 Supplementary Notes

4.1 Supplementary Note 1: Evaluation of $\mathrm{COSMO-CLM}^2$ performance

Satellite-based land surface temperature (LST) datasets are commonly employed to examine the impacts of land use change. A previous study[4] utilized the Global Land Surface Satellite (GLASS) LST product, spanning 2002-2012, to compare adjacent forest land units with open land (grassland and cropland). In the present study, we focus on temperature data from forest and grassland land use tiles and compare the simulated LST differences between these land types within the same grid cells. The results (Figure S27) indicate that the model successfully reproduces the spatial pattern of the annual mean daily maximum LST difference, but fails to simulate the significant difference observed in the annual mean daily minimum LST. Another study[5], based on the Moderate Resolution Imaging Spectroradiometer (MODIS) LST product (2008-2012), employed a moving-window algorithm to establish a linear relationship between forest cover and LST feedback, and quantified the maximum potential impact of forest cover change or tree species transition. In this study, we compare the differences between the simulations Def and Aff, representing deforestation (forest replaced by grassland and cropland, Figure S28), and between Afforestation plus transition to coniferous trees (AfN) and AfB (Figure S29), representing forest type transition (from deciduous broadleaf to evergreen coniferous forests). Comparison shows that both results agree on the warming effect of deforestation on summer maximum LST and the cooling effect on spring minimum LST. Regarding tree species transition, although there is a limited number of valid grid cells in the observation-based dataset, the majority of grid cells show a warming effect on maximum LST in summer when transitioning from broadleaf to coniferous forests, which shares the signal with simulations.

In addition to directly comparing LST, an alternative approach to assessing the impacts of land use and land management change involves utilizing a surface energy balance algorithm, which leverages multiple satellite observations of land properties and energy fluxes[6]. This method enables the calculation of the radiative impacts of forest changes. By comparing the LST differences between coniferous forests, broadleaf forests, and grassland in the Ctl simulation, we can assess the agreement with the dataset from[6]. The results (Figure S30 and S31) indicate that the model successfully replicates the cooling effects of afforestation (both coniferous and broadleaf) in low-latitude regions during the summer, and the warming effect associated with the transition from broadleaf to coniferous forests is also well captured. Given that LST is highly correlated with surface air temperature[3], we are confident in the signal of the BGP impacts simulated by COSMO-CLM^2, particularly for the mean daily mean and maximum temperatures of the summer.

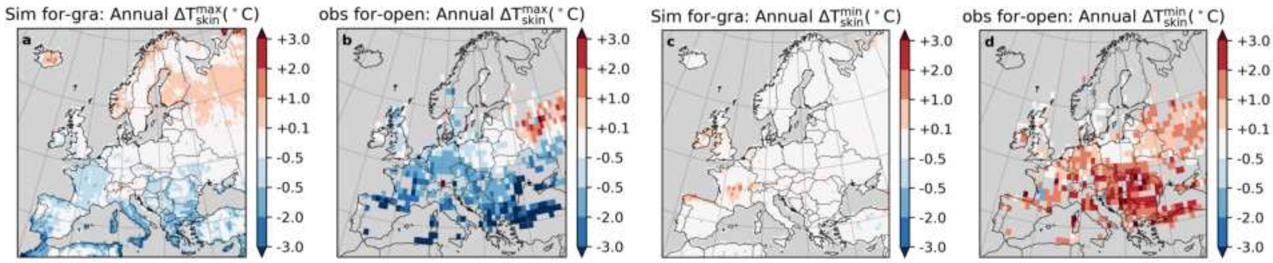

Supplementary Figure S27 | a,c Simulated difference in multi-year (2025-2059) mean daily maximum (T_{skin}^{max}) and daily minimum land surface temperature (T_{skin}^{min}) between the forest and grassland land-use tile in the control simulation. **b,d** Observation-based difference in multi-year (2002-2012) mean T_{skin}^{max} and T_{skin}^{min}[4].

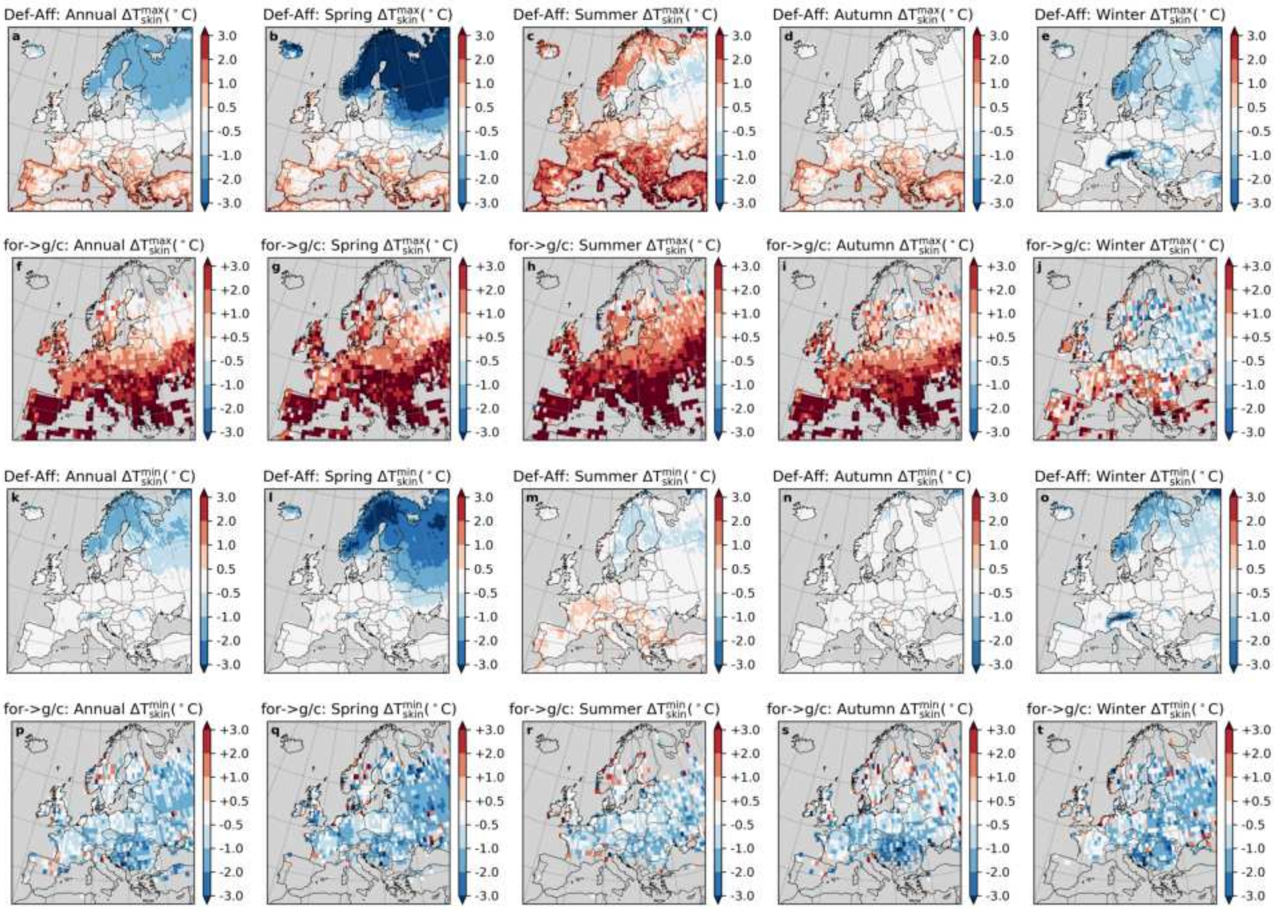

Supplementary Figure S28 | a-e, k-o Simulated difference in multi-year (2025-2059) mean daily maximum (T_{skin}^{max}) and daily minimum land surface temperature (T_{skin}^{min}) between the forestation and deforestation scenarios (Def-Aff). **b-f,p-t** Observation-based potential of changes in multi-year (2008-2012) mean T_{skin}^{max} and T_{skin}^{min} because of deforestation[5].

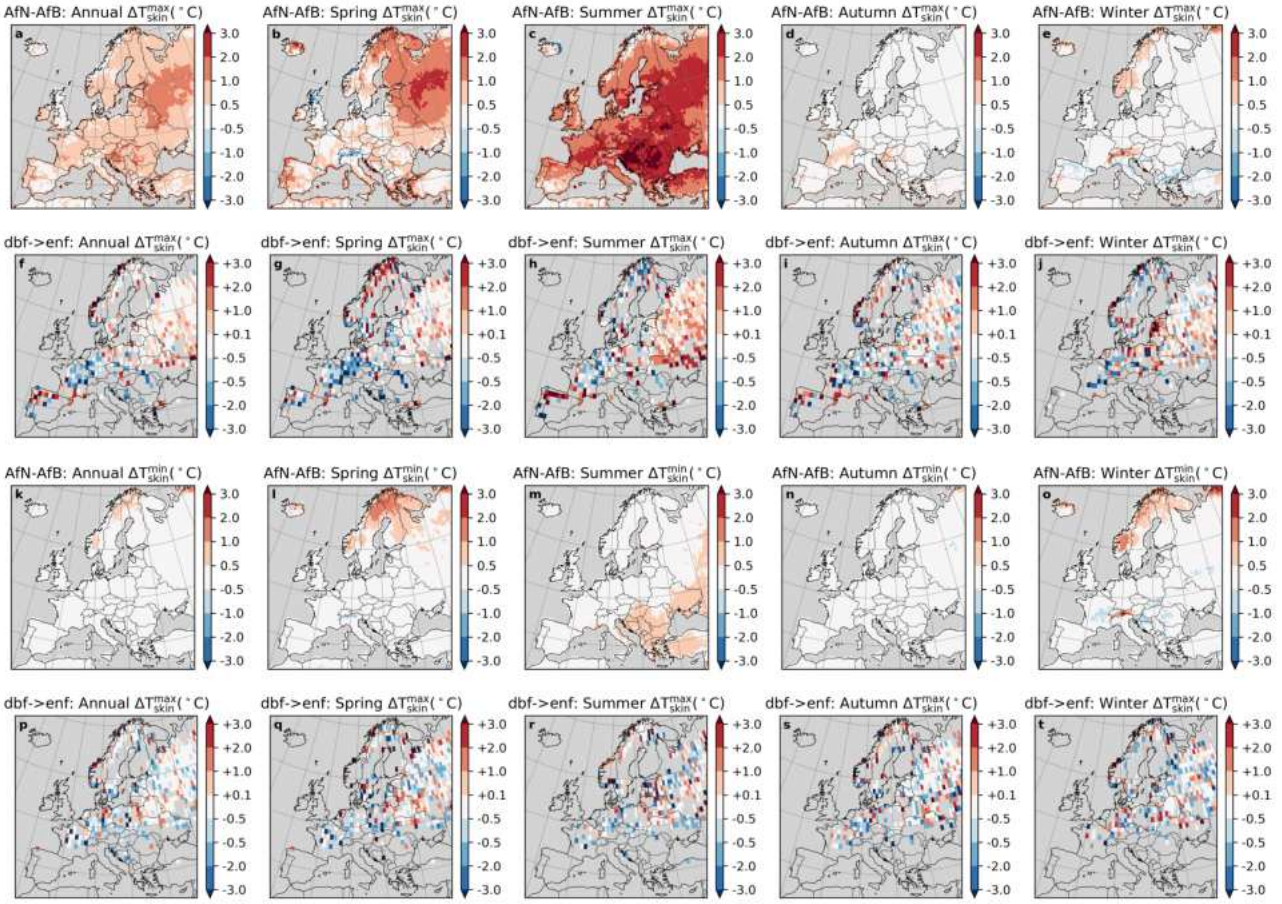

Supplementary Figure S29 | **a-e, k-o** Simulated difference in multi-year (2025-2059) mean daily maximum (T^{max}_{skin}) and daily minimum land surface temperature (T^{min}_{skin}) between the combining scenario of forestation and transition from broadleaf to coniferous forests and the opposite transition (AfN-AfB). **b-f,p-t** Observation-based potential of changes in multi-year (2008-2012) mean T^{max}_{skin} and T^{min}_{skin} because of the transition from broadleaf to coniferous forests[5].

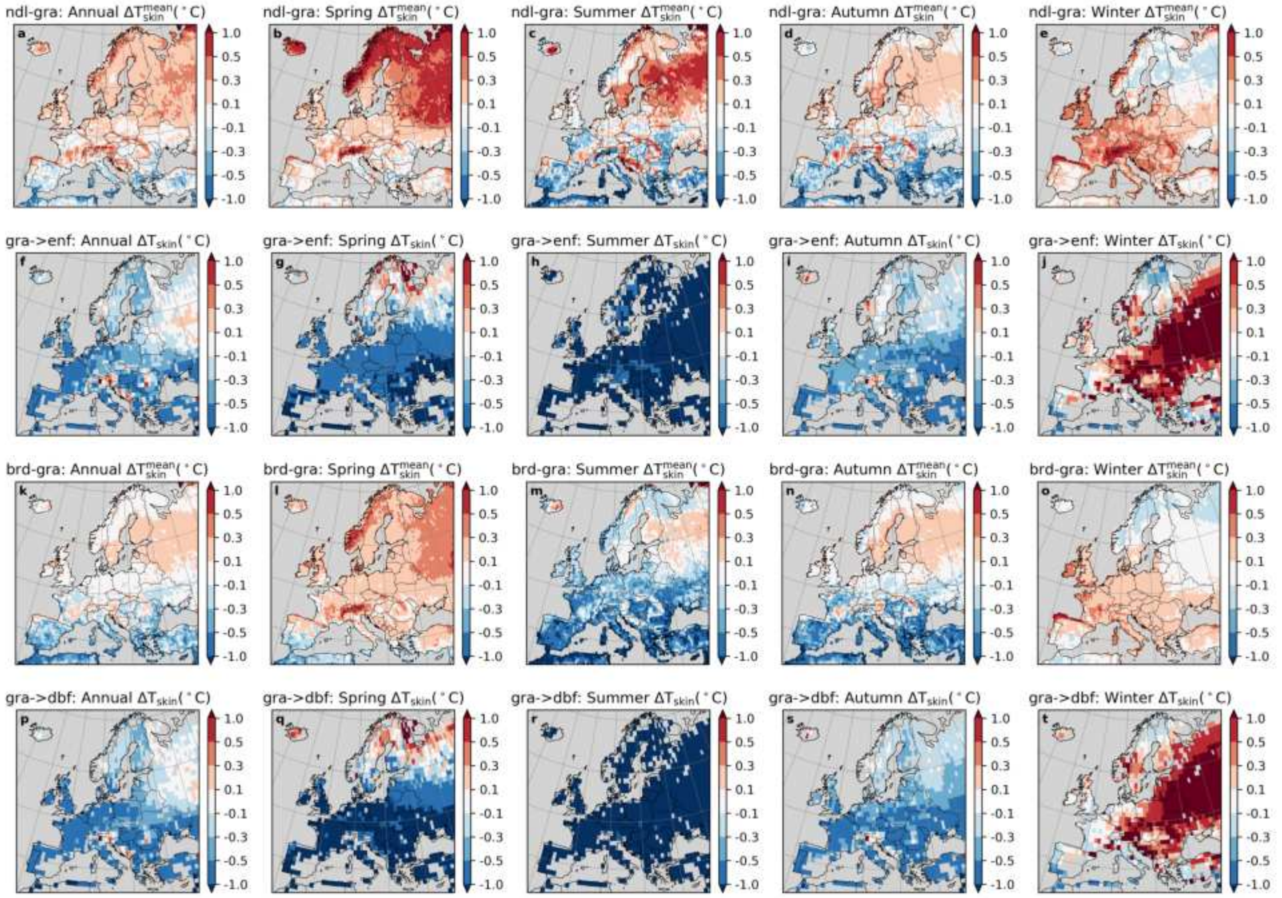

Supplementary Figure S30 | a-e, k-o Simulated difference in multi-year (2025-2059) mean daily maximum (T_{skin}^{max}) and daily minimum land surface temperature (T_{skin}^{min}) between the coniferous forest and grassland land-use tiles, and between the broadleaf forest and grassland land-use tiles. **b-f,p-t** Calculated radiative impacts of the land-use change from grassland to coniferous or broadleaf forest[6].

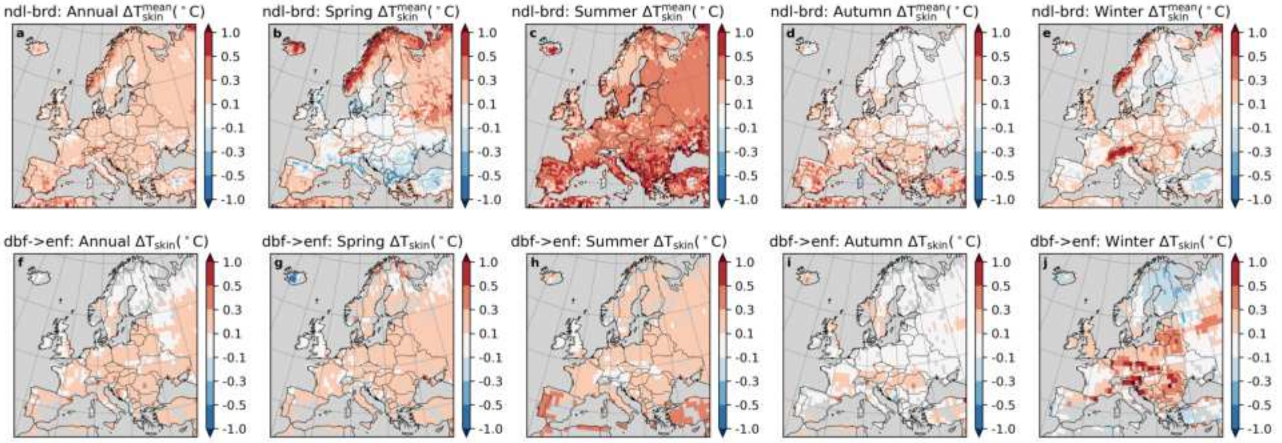

Supplementary Figure S31 | a-e Simulated difference in multi-year (2025-2059) mean daily maximum (T_{skin}^{max}) and daily minimum land surface temperature (T_{skin}^{min}) between the broadleaf and coniferous land-use tiles. **b-f** Calculated radiative impacts of the land-use change from broadleaf to coniferous forest[6].

4.2 Supplementary Note 2: Results from additional sensitivity tests

Compared to land-use change, modifying canopy height or LAI index alone has less impact on temperature (Figure S32). Increasing canopy height slightly increases T_{sumM}, which could be due to the increased roughness, while increased LAI provides more evaporative cooling impacts in summer. Interestingly, Decreasing LAI in spring shows cooling effects in high-latitude areas, which could be due to albedo changes. However, in this study, the forest health scenarios are relatively conservative (multiplied by 1.5 or divided by 1.5), and more extreme scenarios may have more pronounced impacts. Please note that these scenarios are highly idealised and may not reflect the possible changes in reality.

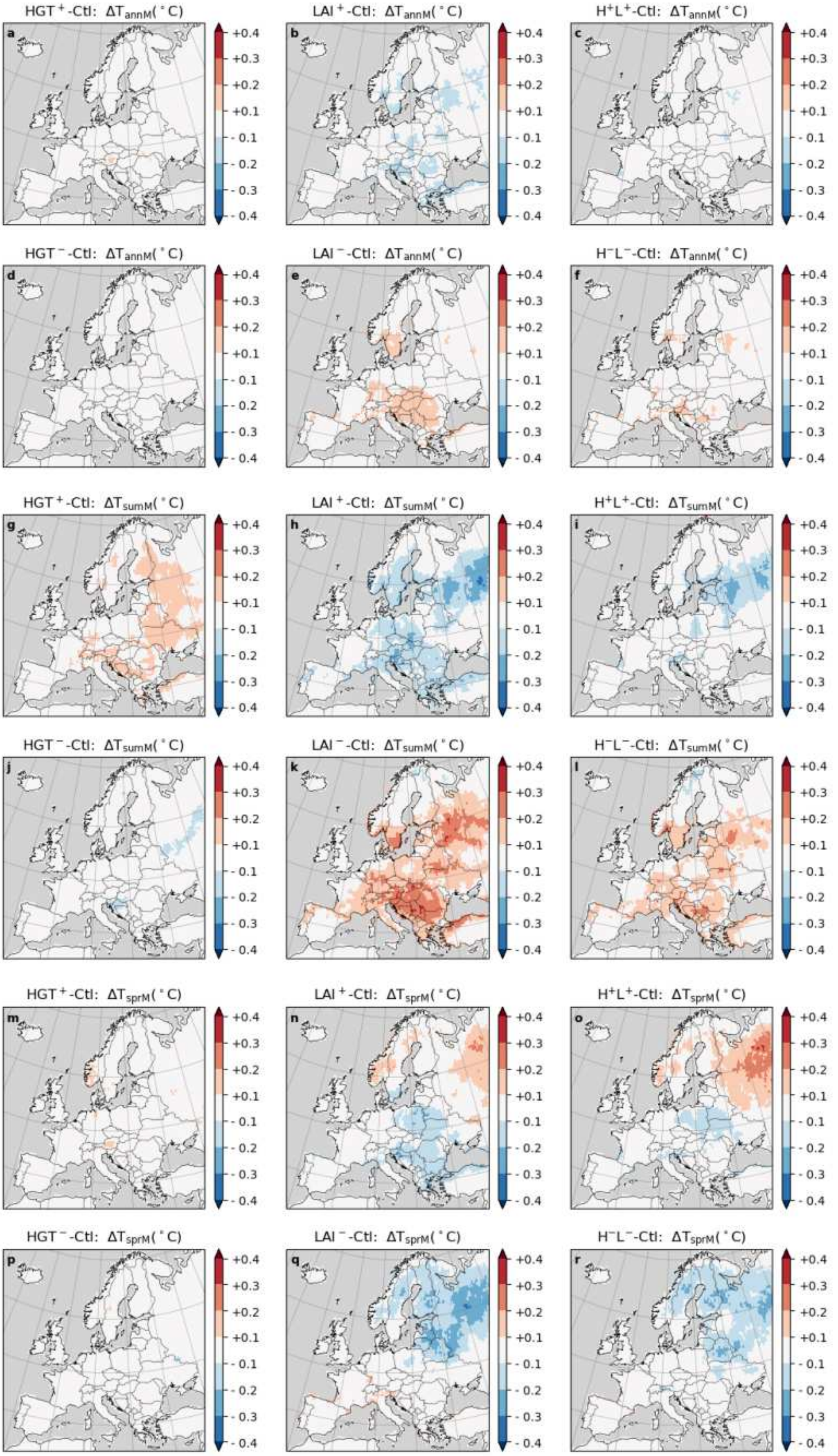

Supplementary Figure S32 | Changes in annual (T_{annM}), summer (T_{sumM}), and spring (T_{sprM}) mean air temperature by changes in canopy height and leaf area index (HGT$^+$: Canopy heights multiplied by 1.5; HGT$^-$: Canopy heights divided by 1.5; LAI$^+$: LAI multiplied by 1.5; LAI$^-$: LAI divided by 1.5; H$^+$L$^+$: both canopy heights and LAI multiplied by 1.5; H$^-$L$^-$: both canopy heights and LAI divided by 1.5) scenarios.

Supplementary References

[1] Yan, H., Wang, S., Billesbach, D., Oechel, W., Zhang, J., Meyers, T., Martin, T., Matamala, R., Baldocchi, D., Bohrer, G. & Others Global estimation of evapotranspiration using a leaf area index-based surface energy and water balance model. *Remote Sensing Of Environment.* **124** pp. 581-595 (2012)

[2] Flanner, M., Shell, K., Barlage, M., Perovich, D. & Tschudi, M. Radiative forcing and albedo feedback from the Northern Hemisphere cryosphere between 1979 and 2008. *Nature Geoscience.* **4**, 151-155 (2011)

[3] Good, E., Ghent, D., Bulgin, C. & Remedios, J. A spatiotemporal analysis of the relationship between near-surface air temperature and satellite land surface temperatures using 17 years of data from the ATSR series. *Journal Of Geophysical Research: Atmospheres.* **122**, 9185-9210 (2017)

[4] Li, Y., Zhao, M., Motesharrei, S., Mu, Q., Kalnay, E. & Li, S. Local cooling and warming effects of forests based on satellite observations. *Nature Communications.* **6**, 6603 (2015)

[5] Duveiller, G., Hooker, J. & Cescatti, A. The mark of vegetation change on Earth's surface energy balance. *Nature Communications.* **9**, 679 (2018)

[6] Bright, R., Davin, E., O'Halloran, T., Pongratz, J., Zhao, K. & Cescatti, A. Local temperature response to land cover and management change driven by non-radiative processes. *Nature Climate Change.* **7**, 296-302 (2017)
